# The assessment of the quality of reporting of meta-analyses in diagnostic research: a systematic review

**DOI:** 10.1186/1471-2288-11-163

**Published:** 2011-12-09

**Authors:** Brian H Willis, Muireann Quigley

**Affiliations:** 1 Biostatistics, University of Manchester, Jean McFarlane Building, Oxford Road, Manchester, M13 9PL, United Kingdom; 2 Centre for Social Ethics and Policy, University of Manchester, Williamson Building, Oxford Road, Manchester, M13 9PL, United Kingdom

## Abstract

**Background:**

Over the last decade there have been a number of guidelines published, aimed at improving the quality of reporting in published studies and reviews. In systematic reviews this may be measured by their compliance with the PRISMA statement. This review aims to evaluate the quality of reporting in published meta-analyses of diagnostic tests, using the PRISMA statement and establish whether there has been a measurable improvement over time.

**Methods:**

Eight databases were searched for reviews published prior to 31^st ^December 2008. Studies were selected if they evaluated a diagnostic test, measured performance, searched two or more databases, stated the search terms and inclusion criteria, and used a statistical method to summarise a test's performance. Data were extracted on the review characteristics and items of the PRISMA statement. To measure the change in the quality of reporting over time, PRISMA items for two periods of equal duration were compared.

**Results:**

Compliance with the PRISMA statement was generally poor: none of the reviews completely adhered to all 27 checklist items. Of the 236 meta-analyses included following selection: only 2(1%) reported the study protocol; 59(25%) reported the searches used; 76(32%) reported the results of a risk of bias assessment; and 82(35%) reported the abstract as a structured summary. Only 11 studies were published before 2000. Thus, the impact of QUOROM on the quality of reporting was not evaluated. However, the periods 2001-2004 and 2005-2008 (covering 93% of studies) were compared using relative risks (RR). There was an increase in the proportion of reviews reporting on five PRISMA items: eligibility criteria (RR 1.13, 95% CI 1.00 - 1.27); risk of bias across studies (methods) (RR 1.81, 95% CI 1.34 - 2.44); study selection results (RR 1.48, 95% CI 1.05 - 2.09); results of individual studies (RR 1.37, 95% CI 1.09 - 1.72); risk of bias across studies (results) (RR 1.65, 95% CI 1.20 - 2.25).

**Conclusion:**

Although there has been an improvement in the quality of meta-analyses in diagnostic research, there are still many deficiencies in the reporting which future reviewers need to address if readers are to trust the validity of the reported findings.

## Background

Systematic reviews have become increasingly important in diagnostic research [1,2]. With the development of new statistical methods used to aggregate primary studies [3,4], and increasing numbers of diagnostic reviews appearing in the literature [1,2], the need for high quality meta-analyses of diagnostic tests has, perhaps, never been greater.

Unfortunately, like all other types of systematic reviews, they are prone to a number of shortcomings. These may arise at a review level, due to inaccessibility to all pertinent studies [5], failings in the selection process [6], or heterogeneity [7,8], which often blights reviews of diagnostic tests [2]. They may also arise at a primary study level, due to flaws in the design, execution and reporting of the component studies [9,10].

To help identify and mitigate potential weaknesses, quality assessment of the primary studies has become an intrinsic element of the review process [11]. Following the publication of the Standards for the Reporting of Diagnostic accuracy studies (STARD) statement [10], which set out to improve the reporting of primary research on diagnostic tests, the assessment of quality has been recently formalized. The Quality Assessment of Diagnostic Accuracy Studies (QUADAS) tool [12] is a generic tool that covers the major domains affecting diagnostic study validity, thus placing quality assessment on a firmer ground and allowing inter-study comparison.

There have been parallel developments in meta-analysis. As major undertakings of work, their results may be influential to health care providers, researchers, and decision makers. Thus, the need for a consistent framework of reporting was recognised. This led to the compilation of the Quality of Reporting of Meta-analyses (QUOROM) statement [13], which was aimed at improving the quality of published meta-analyses of randomised controlled trials. Recently, the QUOROM statement [13] has been superseded by the Preferred Reporting Items for Systematic Reviews and Meta-Analyses (PRISMA) statement [14]. This was in response to developments in systematic review methodology and to widen the scope beyond randomised controlled trials. Currently this is the standard for investigators when reporting their findings and also provides a benchmark by which meta-analyses may be appraised.

Meta-analyses of diagnostic tests will potentially have an increasing role in healthcare as decision makers look to the evidence before implementing new diagnostic technologies. It is important that such analyses provide reliable results and this, in part, is determined by the quality of reporting [2,14,15].

As meta-analysis in diagnostic research has been developing for nearly two decades, it is an appropriate time to assess the overall quality of reporting of meta-analyses of diagnostic test studies. Furthermore it is of interest to know whether there has been a measureable improvement in the quality of reporting. Thus, the objective of this systematic review was to examine the quality of reporting of published meta-analyses of diagnostic tests studies, by their compliance with the PRISMA statement and to assess whether there is evidence of an improvement in the overall quality of reporting. This review was part of a wider investigation into meta-analyses of diagnostic test accuracy studies that has been published elsewhere [16].

## Methods

### Data sources and searches

The electronic databases, Medline, CINAHL, Cochrane library (including the Cochrane Database of Systematic Reviews, DARE, Health Technology Assessment Database and NHS Economic Evaluation Database) EMBASE, PsychInfo, Global health, HMIC, and AMED were all searched for relevant reviews (example search algorithms are listed in Additional file 1). The searches were conducted initially in September 2008 and updated in September 2009. The cut off for inclusion of the meta-analyses was December 31^st ^2008.

### Selection criteria

For the purpose of this review, the term 'meta-analysis' is taken to mean a special type of systematic review, in which standard systematic review methodology has been followed and a quantitative summary of the results has been derived.

All citations retrieved from the electronic searches were subject to a six-step algorithm for inclusion in the review. The title and abstracts of the citations were initially screened using step 1, before retrieving the full text. Steps 2 to 6 were then applied to the full text of the articles, where non-compliance with any of the steps resulted in the article's exclusion.

The steps in the inclusion criteria were as follows:

1. Is the citation an original study of a diagnostic/screening test?

2. Was one of the objectives to measure the test(s) performance?

3. Were two or more of the major databases searched to identify the relevant articles? Examples included MEDLINE, EMBASE, Cochrane, and CINAHL.

4. Were the search terms explicitly stated?

5. Were the inclusion criteria explicitly stated?

6. Was at least one statistical method used to summarize the overall test performance across the primary studies?

The first criterion (step 1) requires clarification as it encompasses a number of terms. The word original was defined here as a primary or secondary (systematic review) evaluation of the technology. Narrative reviews, editorials and commentaries were excluded, although primary studies were still included at this stage. A diagnostic/screening test was defined as a technology aimed at identifying a target disorder, which was present at the time of testing. Target disorders were considered to be pathological processes and not related to a success or failure of an intervention, such as successful placing of stents. Furthermore, technologies, which predicted the future occurrence of a target disorder, which was not present at the time of testing, were not considered diagnostic technologies in this review.

The question of what constitutes a systematic review is, to a degree, open to debate. But the view taken here is that, an important part of the systematic review process is that investigators should make every effort to identify *all *the relevant studies. Since research has demonstrated that to search a single database runs a high risk of missing relevant studies [17,18], a minimum requirement that investigators should have searched two or more databases was imposed in the inclusion criteria.

The first author (BHW) screened and applied the inclusion criteria to all the citations and reviews. The second author (MQ) independently screened and applied the inclusion criteria to a random sample of 10% of all the citations, and discrepancies were decided by consensus agreement.

### Data extraction and quality assessment

Data were abstracted on the following items: publication year; objective; diagnostic test; target disorder; search terms (or whether an algorithm was given); databases searched; inclusion criteria (and whether they were made explicit); process of data extraction; method used to assess quality including QUADAS [12]; presence of heterogeneity; and responses to the PRISMA statement [14].

The PRISMA statement was used to evaluate the overall quality of reporting of the meta-analyses and consists of a twenty seven-point checklist. To indicate the degree of compliance, each checklist item was assigned one of three responses: 'yes' for total compliance; 'partial' for partial compliance; and 'no' for non-compliance. As a large number of checklist items may not be satisfactorily answered with a binary response (yes/no), the intermediate category (partial) was included to represent the situation where a review had satisfied some, but not all of the criteria for an individual item.

Extraction of data was performed by BHW and independently on a random sample of 10%, by MQ. Discrepancies were decided by consensus agreement.

### Data synthesis and analysis

For making comparisons over time, two cohorts of equal duration were compared.

Ideally, these would have been periods either side of the introduction of the QUOROM statement (published in November 1999) so that the impact it had on the quality of reporting could be evaluated. Unfortunately, the sample size of studies prior to its introduction was too small to address this question adequately. Hence, both periods were chosen to be later than the publication of QUOROM to avoid it having a heterogeneous effect on one of the cohorts (see Results).

When analysing responses to the PRISMA statement [14], the relative risk or risk ratio was used as the summary statistic for sub-group comparisons [19,20]. If the relative risk was undefined, then Fisher's exact test was used [21]. Statistical significance was set at p < 0.05. Where appropriate, the kappa statistic was used to assess the level of agreement between the reviewers [22,23], and interpretation was made using accepted criteria [23,24]. For all statistical analyses, EXCEL and the programming software R (version 2.10.1) were used.

## Results

**Figure 1:**
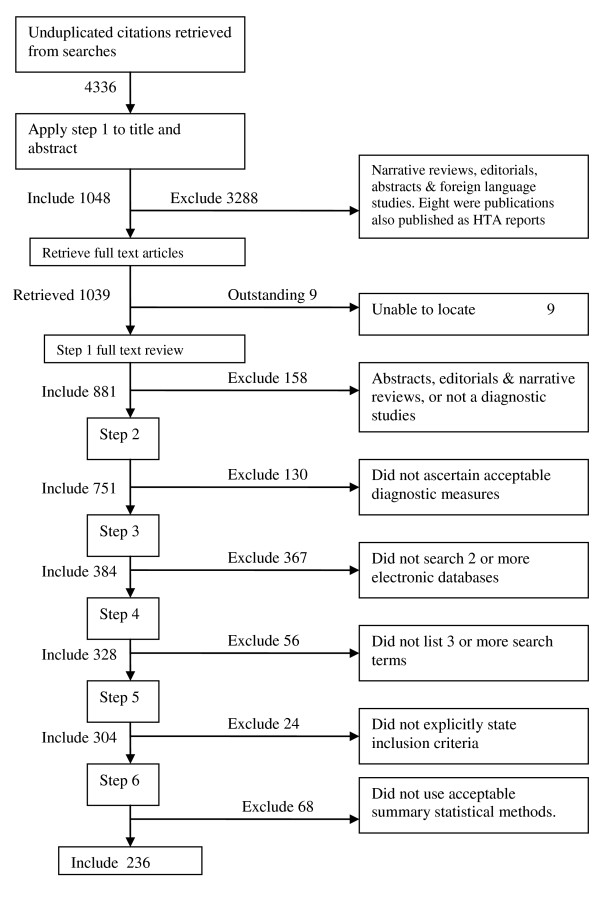
**Flowchart of studies showing results of applying the inclusion criteria**. Also shown are the types of study or reasons for exclusion.

Over 4000 unduplicated citations were retrieved from the electronic searches and after applying the selection criteria 236 articles were included for appraisal (Figure 1). For a list of the included reviews see Additional file 2. As measured by the kappa score, agreement between the reviewers on review selection was excellent, with a kappa score of 0.86. Disagreements were over the stage in which certain reviews were excluded, not the decision whether to exclude. Thus, there was 100% agreement on which reviews to include. For the PRISMA items, the median kappa score between the two reviewers was 0.88 (range: 0.66 - 1).

### Characteristics of the included reviews

The majority of the included reviews were reported in specialist journals (78%), with 39 (17%) being published in radiology journals. Thirty meta-analyses were reported in general medical journals, such as the BMJ or Annals of Internal Medicine and 15 were commissioned health technology assessments (HTA).

**Figure 2:**
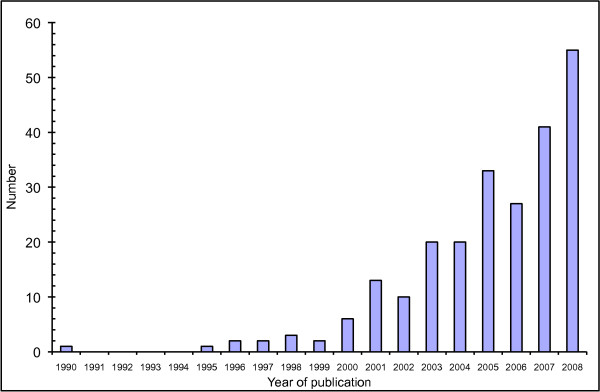
**Number of included meta-analyses per year of publication**.

Figure 2 illustrates the number of reviews per publication year in the included set. Nearly 93% (219 meta-analyses) were published after the year 2001.

A wide range of diagnostic tests featured in the reviews, with imaging technologies (47%) being the most common category of test evaluated. These included 45 reviews on ultrasound tests, 42 on computer tomography and 34 on magnetic resonance imaging. Nearly a fifth of reviews investigated diagnostic tests found in the clinical examination and 35 (15%) evaluated biochemical tests.

An equally diverse spectrum of target disorders was covered by the reviews. Cancer (25%) and infection (20%) were the two most frequent categories of target disorder in the reviews; 20 reviews evaluated diagnostic tests used to detect tuberculosis alone (the characteristics of the individual reviews are detailed elsewhere [16]).

### Quality of reporting and compliance with PRISMA (table 1)

Generally compliance with PRISMA was poor: none of the 27 checklist criteria was complied with by all reviews, nor did any one review fulfil all 27 items of PRISMA.

**Table 1:** Summary of the results of applying the PRISMA statement to the individual meta-analyses

PRISMA statement results								
			**Yes**		**Partial**		**No**	

**Title**	1	Title	221	(93%)	13	(5%)	2	(0%)

**Abstract**	2	Structured summary	82	(35%)	147	(62%)	7	(3%)

**Introduction**	3	Rationale	233	(98%)	3	(1%)	0	(0%)

**Methods**	4	Objectives	143	(60%)	90	(38%)	3	(1%)
	5	Protocol and registration	2	(1%)	35	(15%)	199	(84%)
	6	Eligibility criteria	209	(88%)	27	(11%)	0	(0%)
	7	Information sources	235	(99%)	1	(0%)	0	(0%)
	8	Search	59	(25%)	177	(75%)	0	(0%)
	9	Study selection	134	(57%)	30	(13%)	72	(30%)
	10	Data collection process	149	(63%)	16	(7%)	71	(30%)
	11	Data items	127	(54%)	64	(27%)	45	(19%)
	12	Risk of bias in individual studies	103	(43%)	79	(33%)	54	(23%)
	13	Summary measures	235	(99%)	0	(0%)	1	(0%)
	14	Synthesis of results	234	(99%)	2	(1%)	0	(0%)
	15	Risk of bias across studies	152	(64%)	30	(13%)	54	(23%)
	16	Additional analyses	101	(43%)	12	(5%)	123	(52%)

**Results**	17	Study selection	112	(47%)	91	(38%)	33	(14%)
	18	Study characteristics	132	(56%)	94	(40%)	10	(4%)
	19	Risk of bias within studies	76	(32%)	84	(36%)	76	(32%)
	20	Results of individual studies	162	(68%)	36	(15%)	39	(16%)
	21	Synthesis of results	235	(99%)	1	(0%)	0	(0%)
	22	Risk of bias across studies	136	(57%)	35	(15%)	65	(27%)
	23	Additional analyses	90	(38%)	24	(10%)	122	(51%)

**Discussion**	24	Summary of evidence	232	(98%)	4	(2%)	0	(0%)
	25	Limitations	139	(59%)	38	(16%)	59	(25%)
	26	Conclusions	232	(98%)	4	(2%)	0	(0%)

**Funding**	27	Funding	114	(48%)	0	(0%)	122	(51%)

All of the reviews did at least achieve partial compliance in nearly a third (8/27) of the criteria.

However, some of these items relate to the definition of a systematic review and coincide with the eligibility criteria for this study. Thus a review's partial compliance with PRISMA items 6,7,8 and 14 follows directly from its eligibility for inclusion in this study. Full details of applying the PRISMA statement to each of the included meta-analyses are given in Additional file 3.

There were individual PRISMA items which were adequately reported in over 95% of the reviews and these included: the rationale for the review; the description of the data sources; the measures used to summarize the primary studies; the methods used to aggregate the data; the results of the meta-analysis; a summary of the main findings and the concluding remarks in the discussion.

In over 84% (199/236) of publications the existence of a review protocol, or whether the review had been registered, was not reported. The test being investigated was usually stated in the objective, but, in general, the reporting in the included reviews fell short of stating clear focused objectives. In six reviews the objective was not clear at all (see Additional file 4)

Search algorithms used to locate the primary studies were reported in only 23% (55/236) of the meta-analyses. In a number of the included reviews, the reference lists of the primary studies were searched, for further citations. Nevertheless, in over a third, the searches were confined to only two electronic databases, principally Medline and EMBASE, thereby diminishing the likelihood of achieving completeness (see Additional file 4).

In all of the reviews, the eligibility criteria were explicitly stated in the methods; however, there was significant variability in the clarity of terminology and in only 67 (28%) reviews could they be feasibly described as being algorithmic. The process of selection was also variable: in only 96 (41%) reviews was it clearly described that at least two investigators had independently screened, selected and abstracted data from the primary studies (see Additional file 4).

From the PRISMA analysis, screening and eligibility results were displayed in the form of a flow chart in 113 (48%) reviews. However, on closer inspection, only 44 (19%) of these meta-analyses gave sufficient information on the decisions behind including or excluding the primary studies. Either the reason was not given, or vague terms, such as 'not relevant', were used without elaboration (see Additional file 4).

The descriptions of data abstraction also varied widely. In 103 (43%) reviews, the authors were unclear on either the abstraction process or the data items abstracted (Additional file 4). Many reviews listed no more than abstracting data to complete 2 × 2 tables, when clearly other information had been sought from the primary studies. In eighteen reviews, data abstraction was not reported at all.

For more than half of the reviews (123/236), additional analyses in the methods, such as meta-regression or sub-group analyses, were not described. In a similar proportion there was neither reporting of the results of any additional analyses (52%) nor whether the review had been funded (52%).

### Comparison of meta-analyses published between 2001-2004 and 2005-2008

To determine whether there had been an improvement in the quality of reporting over time, cohorts of meta-analyses published over periods of similar duration were compared. The question of what impact the QUOROM statement had on the quality of reporting was not addressed, owing to the small sample size of the studies published before QUOROM (11 included studies).

Therefore, the period 2001-2004 (63 reviews) was compared with 2005-2008 (156 reviews) for full compliance in each of the PRISMA criteria. Although not pre-specified at the inception stage of the review, the periods 2001-2004 and 2005-2008 were chosen as they not only capture the vast majority of reviews, both are later than the publication of the QUOROM statement (November 1999). The advantage of this latter point is that the earlier cohort does not contain studies published both pre and post publication of QUOROM and so is less likely to be heterogeneous.

**Figure 3:**
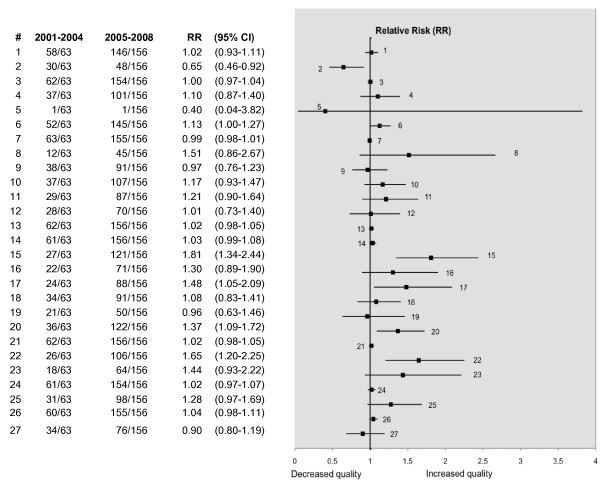
**Comparison of periods 2001-04 and 2005-08 by compliance with the PRISMA statement**. The numbered items (#) correspond to the PRISMA item numbers (see table 1). RR (95% CI) denotes the relative risk with the associated 95% confidence interval.

**Figure 4:**
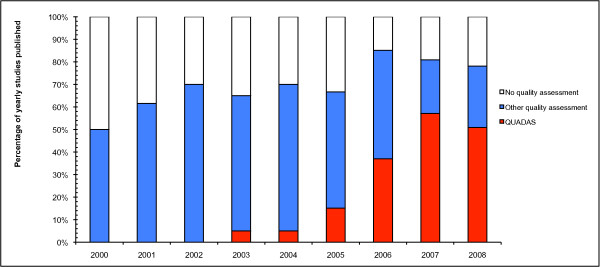
**Changing pattern of quality assessment in meta-analyses of diagnostic tests**. Comparison of the percentage of reviews published per year using the QUADAS tool, other forms of quality assessment and no quality assessment. Earlier years not included due small sample sizes (around 2 studies per year).

Over the two periods, there was a significant improvement demonstrated in five of the criteria and these are illustrated in Figure 3. In only one of the items (abstract) was the reporting poorer in the later period (Figure 3). In the previous decade there also seems to have been an improvement in the number of reviews reporting on the assessment of the quality of the primary studies. Over 50% of the reviews published in 2000 reported no formal quality assessment of the primary studies, compared with 20% in 2008. This has coincided with a greater number of investigators using the QUADAS tool for quality assessment, although other methods of quality assessment continue to be used (see Figure 4).

As already noted, some items of the PRISMA statement feature in the eligibility criteria for this review. The effect is to potentially increase compliance with these items across both periods and bias the relative risk towards 1. This explains the relative risks and narrow confidence intervals observed in items 7 and 14 of Figure 3.

### Comparison of HTA reports with other meta-analyses

**Figure 5:**
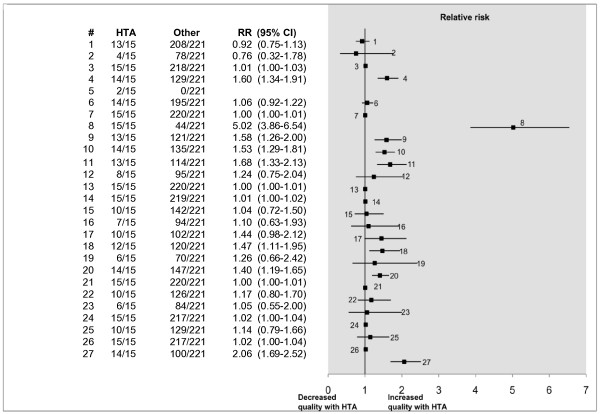
**Comparison of HTA reviews with other reviews using PRISMA**. In nine PRISMA items the HTA reviews were significantly better reported than in other types of reviews. The numbered items (#) correspond to the PRISMA item numbers (see table 1). In item #5, the relative risk was undefined, but, Fisher's exact test demonstrated a significant difference, (p = 0.0038), in favour of the HTA reports. RR (95% CI) denotes the relative risk with the associated 95% confidence interval.

In the included reviews there were 15 Health technology assessment (HTA) reports. These are often commissioned reports conducted by experienced reviewers with a remit of providing 'high quality research information for decision makers' [25] and are not constrained by the word count restrictions imposed by many journals. As might be expected, the reporting in these was of a higher quality. In nine of the PRISMA criteria, there was a significantly higher proportion of HTA reviews adequately reporting on these, compared with the other meta-analyses (see Figure 5). For the individual results of applying PRISMA, refer to Additional file 3.

### Sensitivity analysis

The HTA reports could potentially confound the differences in the quality observed between years 2001-2004 and 2005-2008, particularly if a disproportionate number were in the second cohort (2005-2008). There were 10 HTA reports published between years 2005-2008, and 4 published between 2001-2004 (ratio of 2.5:1). Thus, compared with the overall distribution of meta-analyses, (156 reviews published between 2005-2008 and 63 published between 2001-2004, a ratio of 2.47:1) there were a similar proportion of HTA reports in the later cohort.

**Figure 6:**
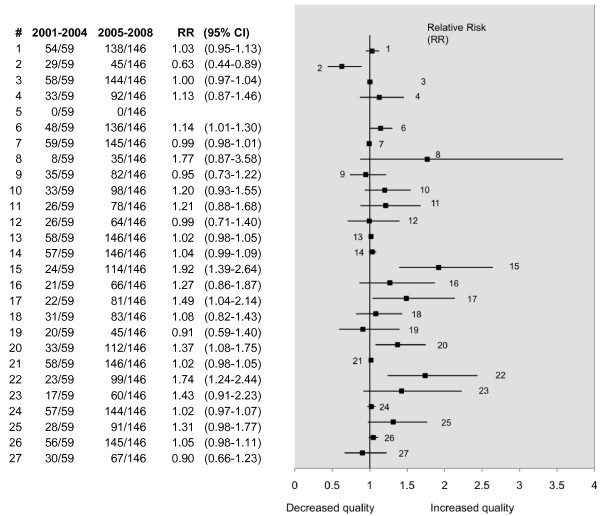
**Sensitivity analysis**. The HTA reports have been removed from the sample to check robustness of results. The numbered items (#) correspond to the PRISMA item numbers (see table 1). In item #5, the relative risk was undefined, but, Fisher's exact test demonstrated no significant difference, (p = 1.00), between the two periods. Overall there was no change in the significance of results in any of the 27 PRISMA items. RR (95% CI) denotes the relative risk with the associated 95% confidence interval.

To test whether this affected the results, a sensitivity analysis was conducted, where the HTA reports were excluded from the data and the analysis repeated. Despite the exclusion of the HTA reports, the results remained robust and the only significant PRISMA items were those in the earlier analysis, that is, items 2, 6, 15, 17, 20 and 22 (Figure 6).

## Discussion

### Summary of evidence

The number of meta-analyses in diagnostic research is increasing annually (Figure 2). From this review it seems that, not only are investigators assessing the quality of primary studies more often (Figure 4), but the quality of reporting of the meta-analyses is also improving. A large part of this is likely to result from the publishing of a number of guidelines over the last decade. The introduction of STARD [10], for primary studies on diagnostic tests, was mirrored by QUOROM [13] and then later PRISMA [14], for systematic reviews and meta-analyses. These have undoubtedly helped standardise the quality of reporting.

The need for high quality studies in health care, which include meta-analyses of diagnostic tests, has probably never been greater as the emphasis on evidence-based decision-making increases. A corollary of this is a drive for more complete and transparent reporting of how a review has been designed and conducted, so that stakeholders may make informed decisions on the validity of the findings [20].

Although the quality of reporting of randomised controlled trials has recently been reported by other authors [20], we are not aware of such an assessment being made on the quality of reporting of published systematic reviews of diagnostic test studies. There was no restriction on the type of journal, test, or target disorders being sought. With a view of capturing those reviews, which were unequivocally meta-analyses that had followed systematic review principles, the inclusion criteria were perhaps tighter than reported in some other reviews [26].

Yet, despite the tighter inclusion criteria, this review found their reporting was, in general, far from adequate and having a number of shortcomings. Using the PRISMA statement [14] it was shown that, although there has been some improvement in certain aspects of reporting over the last decade, for nine PRISMA items less than half the meta-analyses were fully compliant. Some of these may reflect inadequacies in the reporting process, rather than flaws in the design or conduct of the review. Nonetheless, these latter flaws cannot be discounted [27-30]. For example, in 70% of the meta-analyses heterogeneity was reported as being present, yet it was investigated in less than half: it is unlikely that this difference is explained entirely by deficiencies in reporting.

Despite these deficiencies it should be borne in mind that, in addition to the spate of guidelines aimed at improving the quality of reporting [2,10,13], there have been a number of developments in the statistical methodology used in meta-analyses of diagnostic test accuracy studies [3,4,31-33]. Increased dissemination of these methods should lead to increased precision of the summary estimates on a test's performance, which in some instances, should enhance the validity of the reported findings.

### Limitations

There are limitations to this review. Like other recent reviews on the quality of reporting [20], the review process used here, predominately consisted of study selection and data extraction by a single reviewer. A second reviewer performed independent verification of the study selection and data extraction process on a 10% random sample. Although inter-observer agreement demonstrated 'good to excellent agreement' for selection and data extraction, this method is still more likely to yield errors than the preferred method of complete, independent replication of both steps by the two reviewers.

The definition of a systematic review is open to interpretation. Chalmers and Altman described a systematic review as a review, which had been prepared using a 'systematic approach to minimising biases and random errors', with the different components of the process being documented in the 'methods section' [34]. The Cochrane Handbook for Systematic Reviews on Interventions states that systematic reviews possess a number of 'key characteristics' [35]. The eligibility criteria used here were in line with these key characteristics and also coincide with those items of PRISMA that help define a systematic review. It follows that a review's inclusion guarantees it being at least partially compliant with four items of PRISMA. Thus, the PRISMA statement captures more than the quality of reporting in systematic reviews, it captures the essence of what defines a systematic review.

Clearly, if some of the eligibility criteria are relaxed this would not only lead to additional studies being included it would increase the likelihood of poorer compliance with PRISMA. However, it then raises the question of whether these additional studies are truly systematic reviews if they do not possess some of the 'key characteristics' described by, perhaps, the leading authority on systematic reviews [35]. As a result, the criteria used here ensure that there was no ambiguity on the type of reviews included. It also serves to reinforce the conclusion that the quality of reporting in diagnostic systematic reviews is far from adequate if deficiencies in reporting are still being observed in a 'more selective' and therefore 'higher quality' group of studies.

This review is an analysis of the quality of reporting of meta-analyses of diagnostic test studies published over the previous decade. The issue of actual quality of the reviews, as opposed to the quality of reporting, is more difficult to assess, since the PRISMA statement is not a direct measure of quality. Furthermore it is generic checklist aimed at improving the reporting of all types of systematic reviews and does not contain some of the more specific nuances of diagnostic test reviews.

However, it does contain items on the reporting of the process that lie behind the synthesis of a systematic review, and the robustness of this process certainly contributes to the overall quality. There may still be inaccuracies between the reporting and how the review was actually conducted, as has been shown with some primary studies [27-30], nevertheless, it seems unreasonable not to consider this as providing some measure of the overall quality of the meta-analyses included in this review.

The included reviews represent a diverse group of meta-analyses evaluating a range of diagnostic tests over different settings and target disorders. It is unlikely that quality of reporting will be completely independent of variation in these and so the effect heterogeneity has on the results needs to be considered.

### Conclusion

In summary, this review demonstrates that the quality of reporting of meta-analyses has measurably improved over the previous decade. Unfortunately, there are still many deficiencies, identified in the reporting of meta-analyses of diagnostic test studies, which have been highlighted. These need to be addressed by future investigators, if informed judgements on the validity and reproducibility of the findings of their reviews are to be made.

## Competing interests

The authors declare that they have no competing interests.

## Authors' contributions

Both BHW and MQ selected, extracted and appraised the data. BHW wrote the first draft. MQ commented on, and edited all drafts. All authors read and approved the final manuscript.

## Pre-publication history

The pre-publication history for this paper can be accessed here:

http://www.biomedcentral.com/1471-2288/11/163/prepub

## Supplementary Material

Additional file 1 **Appendix 1 - Search algorithms**. Click here for file

Additional file 2 **Appendix 2 - Set of included studies**. Click here for file

Additional file 3 **Appendix 3 - PRISMA results for individual reviews**. Click here for file

Additional file 4 **Appendix 4 - Evidence Tables**. Click here for file
