# Supplementary material for: The assessment of the quality of reporting of meta-analyses in diagnostic research: a systematic review
Source: BMC Med Res Methodol. 2011 Dec 9;11:163. doi: 10.1186/1471-2288-11-163 (PMC3258221; doi:10.1186/1471-2288-11-163)
Supplement: Additional file 1 — Appendix 1 - Search algorithms. [file 1471-2288-11-163-S1.DOC]

**Appendix 1. Search algorithms**

|  | **PsychInfo SEARCH** |  |  | **CINAHL SEARCH** |
| --- | --- | --- | --- | --- |
| 1 | exp diagnosis/ |  | 1 | exp "sensitivity and specificity"/ |
| 2 | diagnos$.tw. |  | 2 | exp ODDS RATIO/ |
| 3 | exp SCREENING/ |  | 3 | exp ROC Curve/ |
| 4 | exp SCREENING TESTS/ |  | 4 | (area adj under adj the adj curve).mp. |
| 5 | exp Meta Analysis/ |  | 5 | AUC.mp. |
| 6 | meta-analys$.tw. |  | 6 | (likelihood adj ratio$).mp. |
| 7 | meta-regression.tw. |  | 7 | (receiver adj operat$).mp. |
| 8 | 6 or 7 or 5 |  | 8 | (sensitivit$ and specificit$).mp. |
| 9 | 4 or 1 or 3 or 2 |  | 9 | exp Meta Analysis/ |
| 10 | 8 and 9 |  | 10 | meta-analys$.mp. |
| 11 | (sensitivit$ and specificit$).tw. |  | 11 | meta-regression.mp. |
| 12 | (receiver adj operat$).tw. |  | 12 | exp DIAGNOSIS/ |
| 13 | (odds adj ratio$).tw. |  | 13 | screening$.mp. |
| 14 | (likelihood adj ratio$).tw. |  | 14 | diagnos$.mp. |
| 15 | (area adj under adj the adj curve).tw. |  | 15 | 6 or 3 or 7 or 2 or 8 or 1 or 4 or 5 |
| 16 | exp MEASUREMENT/ |  | 16 | 11 or 10 or 9 |
| 17 | 11 or 16 or 13 or 12 or 15 or 14 |  | 17 | 13 or 12 or 14 |
| 18 | 10 and 17 |  | 18 | 16 and 17 and 15 |

The search algorithms for used to search Medline and EMBASE may be found in [16].
