# Supplementary material for: The assessment of the quality of reporting of meta-analyses in diagnostic research: a systematic review
Source: BMC Med Res Methodol. 2011 Dec 9;11:163. doi: 10.1186/1471-2288-11-163 (PMC3258221; doi:10.1186/1471-2288-11-163)
Supplement: Additional file 2 — Appendix 2 - Set of included studies. [file 1471-2288-11-163-S2.DOC]

**Appendix 2 – Set of included reviews**

| Abdulla J, Abildstrom SZ, Gotzsche O, Christensen E, Kober L, Torp-Pedersen C. 64-multislice detector computed tomography coronary angiography as potential alternative to conventional coronary angiography: a systematic review and meta-analysis. *European Heart Journal* 2007; 28(24):3042-3050. |
| --- |
| Abubakar I, Irvine L, Aldus CF, Wyatt GM, Fordham R, Schelenz S et al. A systematic review of the clinical, public health and cost-effectiveness of rapid diagnostic tests for the detection and identification of bacterial intestinal pathogens in faeces and food. *Health Technology Assessment* 2007; 11(36):1-216. |
| Akcil M, Karaagaoglu E, Demirhan B, Akcil M, Karaagaoglu E, Demirhan B. Diagnostic accuracy of fine-needle aspiration cytology of palpable breast masses: an SROC curve with fixed and random effects linear meta-regression models. *Diagnostic Cytopathology* 2008; 36(5):303-310. |
| Arbyn M, Bergeron C, Klinkhamer P, Martin-Hirsch P, Siebers AG, Bulten J et al. Liquid compared with conventional cervical cytology: a systematic review and meta-analysis. *Obstetrics & Gynecology* 2008; 111(1):167-177. |
| Arbyn M, Buntinx F, Van RM, Paraskevaidis E, Martin-Hirsch P, Dillner J. Virologic versus cytologic triage of women with equivocal Pap smears: a meta-analysis of the accuracy to detect high-grade intraepithelial neoplasia. *Journal of the National Cancer Institute* 2004; 96(4):280-293. |
| Atieh MA, Atieh MA. Accuracy of real-time polymerase chain reaction versus anaerobic culture in detection of Aggregatibacter actinomycetemcomitans and Porphyromonas gingivalis: a meta-analysis. *Journal of Periodontology* 2008; 79(9):1620-1629. |
| Bafounta ML, Beauchet A, Aegerter P, Saiag P. Is dermoscopy (epiluminescence microscopy) useful for the diagnosis of melanoma? Results of a meta-analysis using techniques adapted to the evaluation of diagnostic tests. *Archives of Dermatology* 2001; 137(10):1343-1350. |
| Bafounta ML, Beauchet A, Chagnon S, Saiag P. Ultrasonography or palpation for detection of melanoma nodal invasion: a meta-analysis. *Lancet Oncology* 2004; 5(11):673-680. |
| Bagai A, Thavendiranathan P, Detsky AS. The rational clinical examination. Does this patient have hearing impairment? *JAMA.* 2006; 295(4):416-428. |
| Bakis S, Irwig L, Wood G, Wong D. Exfoliative cytology as a diagnostic test for basal cell carcinoma: a meta-analysis. *British Journal of Dermatology* 2004; 150(5):829-836. |
| Barnes CJ, Pietrobon R, Higgins LD. Does the pulse examination in patients with traumatic knee dislocation predict a surgical arterial injury? A meta-analysis. *Journal of Trauma-Injury Infection & Critical Care* 2002; 53(6):1109-1114. |
| Bastian LA, Nanda K, Hasselblad V, Simel DL. Diagnostic efficiency of home pregnancy test kits. A meta-analysis. *Archives of Family Medicine* 1998; 7(5):465-469. |
| Benjaminse A, Gokeler A, van der Schans CP. Clinical diagnosis of an anterior cruciate ligament rupture: a meta-analysis. *Journal of Orthopaedic & Sports Physical Therapy* 2006; 36(5):267-288. |
| Berner MM, Kriston L, Bentele M, Harter M. The alcohol use disorders identification test for detecting at-risk drinking: a systematic review and meta-analysis. *Journal of Studies on Alcohol and Drugs* 2007; 68(3):461-473. |
| Berry E, Kelly S, Hutton J, Harris KM, Roderick P. A systematic literature review of the spiral and electron beam computed tomography: with particular reference to clinical applications in hepatic lesions, pulmonary embolus and coronary artery disease. *Health Technology Assessment Southampton* 3(18). |
| Berry E, Kelly S, Westwood ME, Davies LM, Gough MJ, Bamford JM et al. The cost-effectiveness of magnetic resonance angiography for carotid artery stenosis and peripheral vascular disease: A systematic review. *Health Technology Assessment* 2002; 6(7). |
| Bipat S, Glas AS, Slors FJ, Zwinderman AH, Bossuyt PM, Stoker J. Rectal cancer: local staging and assessment of lymph node involvement with endoluminal US, CT, and MR imaging--a meta-analysis. *Radiology* 2004; 232(3):773-783. |
| Bipat S, Glas AS, van d, V, Zwinderman AH, Bossuyt PM, Stoker J. Computed tomography and magnetic resonance imaging in staging of uterine cervical carcinoma: a systematic review. *Gynecologic Oncology* 2003; 91(1):59-66. |
| Bipat S, Phoa SS, van Delden OM, Bossuyt PM, Gouma DJ, Lameris JS et al. Ultrasonography, computed tomography and magnetic resonance imaging for diagnosis and determining resectability of pancreatic adenocarcinoma: a meta-analysis. *Journal of Computer Assisted Tomography* 2005; 29(4):438-445. |
| Blacksell SD, Doust JA, Newton PN, Peacock SJ, Day NP, Dondorp AM. A systematic review and meta-analysis of the diagnostic accuracy of rapid immunochromatographic assays for the detection of dengue virus IgM antibodies during acute infection. *Transactions of the Royal Society of Tropical Medicine & Hygiene* 2006; 100(8):775-784. |
| Brealey S, Scally A, Hahn S, Thomas N, Godfrey C, Coomarasamy A. Accuracy of radiographer plain radiograph reporting in clinical practice: a meta-analysis. *Clinical Radiology* 2005; 60(2):232-241. |
| Brown MD, Lau J, Nelson RD, Kline JA. Turbidimetric D-dimer test in the diagnosis of pulmonary embolism: a metaanalysis. *Clinical Chemistry* 2003; 49(11):1846-1853. |
| Brown MD, Rowe BH, Reeves MJ, Bermingham JM, Goldhaber SZ. The accuracy of the enzyme-linked immunosorbent assay D-dimer test in the diagnosis of pulmonary embolism: a meta-analysis. *Annals of Emergency Medicine* 2002; 40(2):133-144. |
| Bruyninckx R, Aertgeerts B, Bruyninckx P, Buntinx F, Bruyninckx R, Aertgeerts B et al. Signs and symptoms in diagnosing acute myocardial infarction and acute coronary syndrome: a diagnostic meta-analysis. *British Journal of General Practice* 2008; 58(547):105-111. |
| Burr JM, Mowatt G, Hernandez R, Siddiqui MAR, Cook J. The clinical effectiveness and cost-effectiveness of screening for open angle glaucoma: a systematic review and economic evaluation. *Health Technology Assessment* 11(41). |
| Campens D, Buntinx F. Selecting the best renal function tests. A meta-analysis of diagnostic studies. *International Journal of Technology Assessment in Health Care* 1997; 13(2):343-356. |
| Castilla-Rilo J, Lopez-Arrieta J, Bermejo-Pareja F, Ruiz M, Sanchez-Sanchez F, Trincado R. Instrumental activities of daily living in the screening of dementia in population studies: a systematic review and meta-analysis. *International Journal of Geriatric Psychiatry* 2007; 22(9):829-836. |
| Cavallazzi R, Nair A, Vasu T, Marik PE, Cavallazzi R, Nair A et al. Natriuretic peptides in acute pulmonary embolism: a systematic review. *Intensive Care Medicine* 2008; 34(12):2147-2156. |
| Cepoiu M, McCusker J, Cole MG, Sewitch M, Belzile E, Ciampi A et al. Recognition of depression by non-psychiatric physicians--a systematic literature review and meta-analysis. *Journal of General Internal Medicine* 2008; 23(1):25-36. |
| Chalco JP, Huicho L, Alamo C, Carreazo NY, Bada CA. Accuracy of clinical pallor in the diagnosis of anaemia in children: a meta-analysis. *BMC Pediatrics* 2005; 5(46):1-13. |
| Chen SC, Bravata DM, Weil E, Olkin I. A comparison of dermatologists' and primary care physicians' accuracy in diagnosing melanoma: a systematic review. *Archives of Dermatology* 2001; 137(12):1627-1634. |
| Chua AE, Ridley LJ, Chua AE, Ridley LJ. Diagnostic accuracy of CT angiography in acute gastrointestinal bleeding. *Journal of Medical Imaging & Radiation Oncology* 2008; 52(4):333-338. |
| Clark TJ, Mann CH, Shah N, Khan KS, Song F, Gupta JK. Accuracy of outpatient endometrial biopsy in the diagnosis of endometrial hyperplasia. *Acta Obstetricia et Gynecologica Scandinavica* 2001; 80(9):784-793. |
| Clark TJ, Voit D, Gupta JK, Hyde C, Song F, Khan KS. Accuracy of hysteroscopy in the diagnosis of endometrial cancer and hyperplasia: A systematic quantitative review. *Journal of the American Medical Association* 2002; 288(13):1610-1621. |
| Clarke CE, Davies P. Systematic review of acute levodopa and apomorphine challenge tests in the diagnosis of idiopathic Parkinson's disease. *Journal of Neurology Neurosurgery and Psychiatry* 2000; 69(5):590-594. |
| Colin C, Lanoir D, Touzet S, Meyaud-Kraemer L, Bailly F, Trepo C et al. Sensitivity and specificity of third-generation hepatitis C virus antibody detection assays: an analysis of the literature. *Journal of Viral Hepatitis* 2001; 8(2):87-95. |
| Dales RE, Stark RM, Raman S. Computed tomography to stage lung cancer. Approaching a controversy using meta-analysis. *American Review of Respiratory Disease* 1990; 141(5:Pt 1):1096-1101. |
| de Bondt RB, Nelemans PJ, Hofman PA, Casselman JW, Kremer B, van Engelshoven JM et al. Detection of lymph node metastases in head and neck cancer: a meta-analysis comparing US, USgFNAC, CT and MR imaging. *European Journal of Radiology* 2007; 64(2):266-272. |
| de Kroon CD, de Bock GH, Dieben SW, Jansen FW. Saline contrast hysterosonography in abnormal uterine bleeding: a systematic review and meta-analysis. *BJOG: An International Journal of Obstetrics & Gynaecology* 2003; 110(10):938-947. |
| Debrey SM, Yu H, Lynch JK, Lovblad KO, Wright VL, Janket SJ et al. Diagnostic accuracy of magnetic resonance angiography for internal carotid artery disease: a systematic review and meta-analysis. *Stroke* 2008; 39(8):2237-2248. |
| Delgado-Bolton RC, Fernandez-Perez C, Gonzalez-Mate A, Carreras JL. Meta-analysis of the performance of 18F-FDG PET in primary tumor detection in unknown primary tumors. *Journal of Nuclear Medicine* 2003; 44(8):1301-1314. |
| Deville WL, van der Windt DA, Dzaferagic A, Bezemer PD, Bouter LM. The test of Lasegue: systematic review of the accuracy in diagnosing herniated discs. *Spine* 2000; 25(9):1140-1147. |
| Deville WL, Yzermans JC, van Duijn NP, Bezemer PD, van der Windt DA, Bouter LM. The urine dipstick test useful to rule out infections. A meta-analysis of the accuracy. *BMC Urology* 2004; 4(4):1-14. |
| Di Fabio RP. Meta-analysis of the sensitivity and specificity of platform posturography. *Archives of Otolaryngology -- Head & Neck Surgery* 1996; 122(2):150-156. |
| Di NM, Squizzato A, Rutjes AW, Buller HR, Zwinderman AH, Bossuyt PM. Diagnostic accuracy of D-dimer test for exclusion of venous thromboembolism: a systematic review. *Journal of Thrombosis & Haemostasis* 2007; 5(2):296-304. |
| Dinh MT, Abad CL, Safdar N, Dinh MT, Abad CL, Safdar N. Diagnostic accuracy of the physical examination and imaging tests for osteomyelitis underlying diabetic foot ulcers: meta-analysis. *Clinical Infectious Diseases* 2008; 47(4):519-527. |
| Dinnes J, Deeks J, Kunst H, Gibson A, Cummins E. A systematic review of rapid diagnostic tests for the detection of tuberculosis infection. *Health Technology Assessment* 11(3) |
| Dinnes J, Loveman E, McIntyre L, Waugh N. The effectiveness of diagnostic tests for the assessment of shoulder pain due to soft tissue disorders: a systematic review. *Health Technology Assessment* 7(29). |
| Dong MJ, Zhao K, Lin XT, Zhao J, Ruan LX, Liu ZF et al. Role of fluorodeoxyglucose-PET versus fluorodeoxyglucose-PET/computed tomography in detection of unknown primary tumor: a meta-analysis of the literature. *Nuclear Medicine Communications* 2008; 29(9):791-802. |
| Doria AS, Moineddin R, Kellenberger CJ, Epelman M, Beyene J, Schuh S et al. US or CT for Diagnosis of Appendicitis in Children and Adults? A Meta-Analysis. *Radiology* 2006; 241(1):83-94. |
| Dubin MG, Ebert CS, Coffey CS, Melroy CT, Sonnenburg RE, Senior BA. Concordance of middle meatal swab and maxillary sinus aspirate in acute and chronic sinusitis: a meta-analysis. *American Journal of Rhinology* 2005; 19(5):462-470. |
| Ebell MH, White LL, Casault T. A systematic review of the history and physical examination to diagnose influenza. *Journal of the American Board of Family Practice* 2004; 17(1):1-5. |
| Engelbrecht MR, Jager GJ, Laheij RJ, Verbeek AL, van Lier HJ, Barentsz JO. Local staging of prostate cancer using magnetic resonance imaging: a meta-analysis. *European Radiology* 2002; 12(9):2294-2302. |
| Ewald B, Attia J. Which test to detect microalbuminuria in diabetic patients? A systematic review. *Australian Family Physician* 571; 33(7):565-567. |
| Ewald B, Ewald D, Thakkinstian A, Attia J, Ewald B, Ewald D et al. Meta-analysis of B type natriuretic peptide and N-terminal pro B natriuretic peptide in the diagnosis of clinical heart failure and population screening for left ventricular systolic dysfunction. *Internal Medicine Journal* 2008; 38(2):101-113. |
| Fancher TL, White RH, Kravitz RL. Combined use of rapid D-dimer testing and estimation of clinical probability in the diagnosis of deep vein thrombosis: Systematic review. *British Medical Journal* 2004; 329(7470):821-824. |
| Fischer BMB, Mortensen J, Hojgaard L. Positron emission tomography in the diagnosis and staging of lung cancer: a systematic, quantitative review. *Lancet Oncology* 2001; 2(11):659-666. |
| Flores LL, Pai M, Colford JM, Jr., Riley LW. In-house nucleic acid amplification tests for the detection of Mycobacterium tuberculosis in sputum specimens: meta-analysis and meta-regression. *BMC Microbiology* 2005; 5:55. |
| Ford AC, Veldhuyzen van Zanten SJ, Rodgers CC, Talley NJ, Vakil NB, Moayyedi P et al. Diagnostic utility of alarm features for colorectal cancer: systematic review and meta-analysis. *Gut* 2008; 57(11):1545-1553. |
| Fraquelli M, Colli A, Casazza G, Paggi S, Colucci A, Massironi S et al. Role of US in detection of Crohn disease: meta-analysis. *Radiology* 2005; 236(1):95-101. |
| Friedrich-Rust M, Ong MF, Martens S, Sarrazin C, Bojunga J, Zeuzem S et al. Performance of transient elastography for the staging of liver fibrosis: a meta-analysis. *Gastroenterology* 2008; 134(4):960-974. |
| Geifman-Holtzman O, Grotegut CA, Gaughan JP. Diagnostic accuracy of noninvasive fetal Rh genotyping from maternal blood--a meta-analysis. *American Journal of Obstetrics & Gynecology* 2006; 195(4):1163-1173. |
| Gisbert JP, de la Morena F, Abraira V. Accuracy of monoclonal stool antigen test for the diagnosis of H. pylori infection: a systematic review and meta-analysis.[erratum appears in Am J Gastroenterol. 2006 Oct;101(10):2445]. *American Journal of Gastroenterology* 2006; 101(8):1921-1930. |
| Glas AS, Roos D, Deutekom M, Zwinderman AH, Bossuyt PM, Kurth KH. Tumor markers in the diagnosis of primary bladder cancer. A systematic review.*Journal of Urology* 2003; 169(6):1975-1982. |
| Goodacre S, Sampson F, Stevenson M, Wailoo A, Sutton A, Thomas S et al. Measurement of the clinical and cost-effectiveness of non-invasive diagnostic testing strategies for deep vein thrombosis. *Health Technology Assessment* 2006; 10(15):1-168. |
| Gordon I, Barkovics M, Pindoria S, Cole TJ, Woolf AS. Primary vesicoureteric reflux as a predictor of renal damage in children hospitalized with urinary tract infection: A systematic review and meta-analysis. *Journal of the American Society of Nephrology* 2003; 14(3):739-744. |
| Goto M, Noguchi Y, Koyama H, Hira K, Shimbo T, Fukui T. Diagnostic value of adenosine deaminase in tuberculous pleural effusion: a meta-analysis. *Annals of Clinical Biochemistry* 2003; 40(Pt:4):374-381. |
| Gould MK, Kuschner WG, Rydzak CE, Maclean CC, Demas AN, Shigemitsu H et al. Test performance of positron emission tomography and computed tomography for mediastinal staging in patients with non-small-cell lung cancer: a meta-analysis. *Annals of Internal Medicine* 2003; 139(11):879-892. |
| Gu P, Huang G, Chen Y, Zhu C, Yuan J, Sheng S. Diagnostic utility of pleural fluid carcinoembryonic antigen and CYFRA 21-1 in patients with pleural effusion: a systematic review and meta-analysis. *Journal of Clinical Laboratory Analysis* 2007; 21(6):398-405. |
| Gupta JK, Chien PF, Voit D, Clark TJ, Khan KS. Ultrasonographic endometrial thickness for diagnosing endometrial pathology in women with postmenopausal bleeding: a meta-analysis. *Acta Obstetricia et Gynecologica Scandinavica* 2002; 81(9):799-816. |
| Hallan S, Asberg A. The accuracy of C-reactive protein in diagnosing acute appendicitis--a meta-analysis. *Scandinavian Journal of Clinical & Laboratory Investigation* 1997; 57(5):373-380. |
| Halligan S, Altman DG, Taylor SA, Mallett S, Deeks JJ, Bartram CI et al. CT colonography in the detection of colorectal polyps and cancer: systematic review, meta-analysis, and proposed minimum data set for study level reporting. *Radiology* 2005; 237(3):893-904. |
| Hamon M, Champ-Rigot L, Morello R, Riddell JW, Hamon M, Hamon M et al. Diagnostic accuracy of in-stent coronary restenosis detection with multislice spiral computed tomography: a meta-analysis. *European Radiology* 2008^b^; 18(2):217-225. |
| Hamon M, Lepage O, Malagutti P, Riddell JW, Morello R, Agostini D et al. Diagnostic performance of 16- and 64-section spiral CT for coronary artery bypass graft assessment: meta-analysis. *Radiology* 2008^a^; 247(3):679-686. |
| Hancock MJ, Maher CG, Latimer J, Spindler MF, McAuley JH, Laslett M et al. Systematic review of tests to identify the disc, SIJ or facet joint as the source of low back pain. *European Spine Journal* 2007; 16(10):1539-1550. |
| Hayashino Y, Goto M, Noguchi Y, Fukui T. Ventilation-perfusion scanning and helical CT in suspected pulmonary embolism: meta-analysis of diagnostic performance. *Radiology* 2005; 234(3):740-748. |
| Hegedus EJ, Cook C, Hasselblad V, Goode A, McCrory DC. Physical examination tests for assessing a torn meniscus in the knee: a systematic review with meta-analysis. *Journal of Orthopaedic & Sports Physical Therapy* 2007; 37(9):541-550. |
| Hegedus EJ, Goode A, Campbell S, Morin A, Tamaddoni M, Moorman CT, III et al. Physical examination tests of the shoulder: a systematic review with meta-analysis of individual tests. *British Journal of Sports Medicine* 2008; 42(2):80-92. |
| Heijenbrok-Kal MH, Fleischmann KE, Hunink MG. Stress echocardiography, stress single-photon-emission computed tomography and electron beam computed tomography for the assessment of coronary artery disease: a meta-analysis of diagnostic performance. *American Heart Journal* 2007 ^a^; 154(3):415-423 |
| Heijenbrok-Kal MH, Kock MC, Hunink MG. Lower extremity arterial disease: multidetector CT angiography meta-analysis. *Radiology* 2007 ^b^; 245(2):433-439. |
| Hobby JL, Tom BD, Bearcroft PW, Dixon AK. Magnetic resonance imaging of the wrist: diagnostic performance statistics. *Clinical Radiology* 2001; 56(1):50-57. |
| Hofman PA, Nelemans P, Kemerink GJ, Wilmink JT. Value of radiological diagnosis of skull fracture in the management of mild head injury: meta-analysis. *Journal of Neurology, Neurosurgery & Psychiatry* 2000; 68(4):416-422. |
| Holmes JF, Gladman A, Chang CH. Performance of abdominal ultrasonography in pediatric blunt trauma patients: a meta-analysis. *Journal of Pediatric Surgery* 2007; 42(9):1588-1594. |
| Holroyd-Leduc JMT. What type of urinary incontinence does this woman have? *JAMA.* 2008; 299(12):1446-1456. |
| Holty JE, Kuschner WG, Gould MK. Accuracy of transbronchial needle aspiration for mediastinal staging of non-small cell lung cancer: a meta-analysis. *Thorax* 2005; 60(11):949-955. |
| Horsthuis K, Bipat S, Bennink RJ, Stoker J, Horsthuis K, Bipat S et al. Inflammatory bowel disease diagnosed with US, MR, scintigraphy, and CT: meta-analysis of prospective studies. *Radiology* 2008; 247(1):64-79. |
| Hovels AM, Heesakkers RA, Adang EM, Jager GJ, Strum S, Hoogeveen YL et al. The diagnostic accuracy of CT and MRI in the staging of pelvic lymph nodes in patients with prostate cancer: a meta-analysis. *Clinical Radiology* 2008; 63(4):387-395. |
| Huicho L, Campos-Sanchez M, Alamo C. Metaanalysis of urine screening tests for determining the risk of urinary tract infection in children. *Pediatric Infectious Disease Journal* 2002; 21(1):1-11. |
| Ioannidis JP, Lau J. 18F-FDG PET for the diagnosis and grading of soft-tissue sarcoma: a meta-analysis. *Journal of Nuclear Medicine* 2003; 44(5):717-724. |
| Jahromi AS, Cina CS, Liu Y, Clase CM. Sensitivity and specificity of color duplex ultrasound measurement in the estimation of internal carotid artery stenosis: a systematic review and meta-analysis. *Journal of Vascular Surgery* 2005; 41(6):962-972. |
| Jiang J, Shi HZ, Liang QL, Qin SM, Qin XJ. Diagnostic value of interferon-gamma in tuberculous pleurisy: a meta-analysis. *Chest* 2007; 131(4):1133-1141. |
| Jones CM, Athanasiou T, Tekkis PP, Malinovski V, Purkayastha S, Haq A et al. Does Doppler echography have a diagnostic role in patency assessment of internal thoracic artery grafts? *European Journal of Cardio-Thoracic Surgery* 2005; 28(5):692-700. |
| Joshi U, Raijmakers PG, Riphagen II, Teule GJ, van LA, Hoekstra OS. Attenuation-corrected vs. nonattenuation-corrected 2-deoxy-2-[F-18]fluoro-D-glucose-positron emission tomography in oncology: a systematic review. *Molecular Imaging & Biology* 2007; 9(3):99-105. |
| Kalantri S, Pai M, Pascopella L, Riley L, Reingold A. Bacteriophage- based tests for the detection of Mycobacterium tuberculosis in clinical specimens: a systematic review and meta- analysis. *BMC Infectious Diseases* 2005; 5(59):1-13. |
| Karassa FB, Afeltra A, Ambrozic A, Chang DM, De KF, Doria A et al. Accuracy of anti-ribosomal P protein antibody testing for the diagnosis of neuropsychiatric systemic lupus erythematosus: an international meta-analysis. *Arthritis & Rheumatism* 2006; 54(1):312-324. |
| Karassa FB, Matsagas MI, Schmidt WA, Ioannidis JP. Meta-analysis: test performance of ultrasonography for giant-cell arteritis. *Annals of Internal Medicine* 2005; 142(5):359-369. |
| Kassai B, Boissel JP, Cucherat M, Sonie S, Shah NR, Leizorovicz A. A systematic review of the accuracy of ultrasound in the diagnosis of deep venous thrombosis in asymptomatic patients. *Thrombosis & Haemostasis* 2004; 91(4):655-666. |
| Kelly S, Harris KM, Berry E, Hutton J, Roderick P, Cullingworth J et al. A systematic review of the staging performance of endoscopic ultrasound in gastro-oesophageal carcinoma. *Gut* 2001; 49(4):534-539. |
| Khunti K, Squire I, Abrams KR, Sutton AJ. Accuracy of a 12-lead electrocardiogram in screening patients with suspected heart failure for open access echocardiography: a systematic review and meta-analysis. *European Journal of Heart Failure* 2004; 6(5):571-576. |
| Koliopoulos G, Arbyn M, Martin-Hirsch P, Kyrgiou M, Prendiville W, Paraskevaidis E. Diagnostic accuracy of human papillomavirus testing in primary cervical screening: a systematic review and meta-analysis of non-randomized studies. *Gynecologic Oncology* 2007; 104(1):232-246. |
| Kraag N, Thijs C, Knipschild P. Dyspepsia - How noisy are gallstones? A meta-analysis of epidemiologic studies of biliary pain, dyspeptic symptoms, and food intolerance. *Scandinavian Journal of Gastroenterology* 1995; 30(5):411-421. |
| Krug BC. Role of PET in the initial staging of cutaneous malignant melanoma: Systematic review. *Radiology* 2008; 249(3):836-844. |
| Kwee TC, Kwee RM, Alavi A, Kwee TC, Kwee RM, Alavi A. FDG-PET for diagnosing prosthetic joint infection: systematic review and metaanalysis. *European Journal of Nuclear Medicine & Molecular Imaging* 2008; 35(11):2122-2132. |
| Kwee TC, Kwee RM. MR angiography in the follow-up of intracranial aneurysms treated with Guglielmi detachable coils: systematic review and meta-analysis. *Neuroradiology* 2007; 49(9):703-713. |
| Lameris W, van RA, Bipat S, Bossuyt PM, Boermeester MA, Stoker J et al. Graded compression ultrasonography and computed tomography in acute colonic diverticulitis: meta-analysis of test accuracy. *European Radiology* 2008; 18(11):2498-2511. |
| Leal YA, Flores LL, Garcia-Cortes LB, Cedillo-Rivera R, Torres J, Leal YA et al. Antibody-based detection tests for the diagnosis of Helicobacter pylori infection in children: a meta-analysis. *PLoS ONE [Electronic Resource]* 2008; 3(11):e3751. |
| Leeflang MM, Debets-Ossenkopp YJ, Visser CE, Scholten RJ, Hooft L, Bijlmer HA et al. Galactomannan detection for invasive aspergillosis in immunocompromized patients. *Cochrane Database of Systematic Reviews* 2008;(4):CD007394. |
| Liang QL, Shi HZ, Qin XJ, Liang XD, Jiang J, Yang HB et al. Diagnostic accuracy of tumour markers for malignant pleural effusion: a meta-analysis. *Thorax* 2008^a^; 63(1):35-41. |
| Liang QL, Shi HZ, Wang K, Qin SM, Qin XJ, Liang QL et al. Diagnostic accuracy of adenosine deaminase in tuberculous pleurisy: a meta-analysis. *Respiratory Medicine* 2008^b^; 102(5):744-754. |
| Ling DI, Flores LL, Riley LW, Pai M, Ling DI, Flores LL et al. Commercial nucleic-acid amplification tests for diagnosis of pulmonary tuberculosis in respiratory specimens: meta-analysis and meta-regression. *PLoS ONE [Electronic Resource]* 2008^a^; 3(2):e1536. |
| Ling DI, Zwerling AA, Pai M, Ling DI, Zwerling AA, Pai M. GenoType MTBDR assays for the diagnosis of multidrug-resistant tuberculosis: a meta-analysis. *European Respiratory Journal* 2008^b^; 32(5):1165-1174. |
| Liu JL, Wyatt JC, Deeks JJ, Clamp S, Keen J, Verde P et al. Systematic reviews of clinical decision tools for acute abdominal pain. *Health Technology Assessment* 2006; 10(47):1-167. |
| Lysakowski C, Walder B, Costanza MC, Tramer MR. Transcranial Doppler versus angiography in patients with vasospasm due to a ruptured cerebral aneurysm: A systematic review. *Stroke* 2001; 32(10):2292-2298. |
| Makrydimas G, Sotiriadis A, Ioannidis JP. Screening performance of first-trimester nuchal translucency for major cardiac defects: a meta-analysis. *American Journal of Obstetrics & Gynecology* 2003; 189(5):1330-1335. |
| Mant J, McManus RJ, Oakes RAL, Delaney BC, Barton PM. Systematic review and modelling of the investigation of acute and chronic chest pain presenting in primary care. *Health Technology Assessment* 8(2) |
| Martin A, Panaiotov S, Portaels F, Hoffner S, Palomino JC, Angeby K et al. The nitrate reductase assay for the rapid detection of isoniazid and rifampicin resistance in Mycobacterium tuberculosis: a systematic review and meta-analysis. *Journal of Antimicrobial Chemotherapy* 2008; 62(1):56-64. |
| Martin A, Portaels F, Palomino JC. Colorimetric redox-indicator methods for the rapid detection of multidrug resistance in Mycobacterium tuberculosis: a systematic review and meta-analysis. *Journal of Antimicrobial Chemotherapy* 2007; 59(2):175-183. |
| Martin JL, Williams KS, Abrams KR, Turner DA, Sutton AJ, Chapple C et al. Systematic review and evaluation of methods of assessing urinary incontinence. *Health Technology Assessment* 2006; 10(6):1-132. |
| Marx A, Pewsner D, Egger M, Nuesch R, Bucher HC, Genton B et al. Meta-analysis: accuracy of rapid tests for malaria in travelers returning from endemic areas. *Annals of Internal Medicine* 2005; 142(10):836-846. |
| Medeiros LR, Rosa DD, Edelweiss MI, Stein AT, Bozzetti MC, Zelmanowicz A et al. Accuracy of frozen-section analysis in the diagnosis of ovarian tumors: a systematic quantitative review. *International Journal of Gynecological Cancer* 2005; 15(2):192-202. |
| Meijer AB, YL O, Geleijns J, Kroft LJ, Meijer AB, Ying L et al. Meta-analysis of 40- and 64-MDCT angiography for assessing coronary artery stenosis. American Journal of Roentgenology. 2008; 191(6):1667-1675. |
| Meserve BB, Cleland JA, Boucher TR, Meserve BB, Cleland JA, Boucher TR. A meta-analysis examining clinical test utilities for assessing meniscal injury. *Clinical Rehabilitation* 2008; 22(2):143-161. |
| Micames CG, McCrory DC, Pavey DA, Jowell PS, Gress FG. Endoscopic ultrasound-guided fine-needle aspiration for non-small cell lung cancer staging: A systematic review and metaanalysis. *Chest* 2007; 131(2):539-548. |
| Mijnhout GS, Hoekstra OS, van Tulder MW, Teule GJ, Deville WL. Systematic review of the diagnostic accuracy of (18)F-fluorodeoxyglucose positron emission tomography in melanoma patients. *Cancer* 2001; 91(8):1530-1542. |
| Mitchell AJ, Mitchell AJ. The clinical significance of subjective memory complaints in the diagnosis of mild cognitive impairment and dementia: a meta-analysis. *International Journal of Geriatric Psychiatry* 2008; 23(11):1191-1202. |
| Mol BW, Bayram N, Lijmer JG, Wiegerinck MA, Bongers MY, van d, V et al. The performance of CA-125 measurement in the detection of endometriosis: a meta-analysis. *Fertility & Sterility* 1998^a^; 70(6):1101-1108. |
| Mol BW, Lijmer JG, Ankum WM, van d, V, Bossuyt PM. The accuracy of single serum progesterone measurement in the diagnosis of ectopic pregnancy: a meta-analysis. *Human Reproduction* 1998^b^; 13(11):3220-3227. |
| Moles DR, Downer MC, Speight PM. Meta-analysis of measures of performance reported in oral cancer and precancer screening studies. *British Dental Journal* 332; 192(6):340-344. |
| Morgan M, Kalantri S, Flores L, Pai M. A commercial line probe assay for the rapid detection of rifampicin resistance in Mycobacterium tuberculosis: a systematic review and meta-analysis. *BMC Infectious Diseases* 2005; 5:62. |
| Morisson P, Neves DD, Morisson P, Neves DD. Evaluation of adenosine deaminase in the diagnosis of pleural tuberculosis: a Brazilian meta-analysis. *Jornal Brasileiro De Pneumologia.* 2008; 34(4):217-224. |
| Mowatt G, Cummins E, Waugh N, Walker S, Cook J, Jia X et al. Systematic review of the clinical effectiveness and cost-effectiveness of 64-slice or higher computed tomography angiography as an alternative to invasive coronary angiography in the investigation of coronary artery disease. *Health Technology Assessment* 2008; 12(17) |
| Mowatt G, Vale L, Brazzelli M, Hernandez R, Murray A. Systematic review of the effectiveness and the cost-effectiveness, and economic evaluation, of myocardial perfusion scintigraphy for the diagnosis and management of angina and myocardial infarction. *Health Technology Assessment* 8(30) |
| Muchow RD, Resnick DK, Abdel MP, Munoz A, Anderson PA, Muchow RD et al. Magnetic resonance imaging (MRI) in the clearance of the cervical spine in blunt trauma: a meta-analysis. *Journal of Trauma-Injury Infection & Critical Care* 2008; 64(1):179-189. |
| Mulhall BP, Veerappan GR, Jackson JL. Meta-analysis: computed tomographic colonography. *Annals of Internal Medicine* 2005; 142(8):635-650. |
| Nallamothu BK, Saint S, Bielak LF, Sonnad SS, Peyser PA, Rubenfire M et al. Electron-beam computed tomography in the diagnosis of coronary artery disease: a meta-analysis. *Archives of Internal Medicine* 2001; 161(6):833-838. |
| Nandalur KR, Dwamena BA, Choudhri AF, Nandalur MR, Carlos RC. Diagnostic performance of stress cardiac magnetic resonance imaging in the detection of coronary artery disease: a meta-analysis. *Journal of the American College of Cardiology* 2007; 50(14):1343-1353. |
| Nandalur KR, Dwamena BA, Choudhri AF, Nandalur SR, Reddy P, Carlos RC et al. Diagnostic performance of positron emission tomography in the detection of coronary artery disease: a meta-analysis. *Academic Radiology* 2008; 15(4):444-451. |
| Nayak S, Olkin I, Liu H, Grabe M, Gould MK, Allen IE et al. Meta-analysis: accuracy of quantitative ultrasound for identifying patients with osteoporosis. *Annals of Internal Medicine* 2006; 144(11):832-841. |
| Niemann T, Kollmann T, Bongartz G, Niemann T, Kollmann T, Bongartz G. Diagnostic performance of low-dose CT for the detection of urolithiasis: a meta-analysis. American Journal of Roentgenology. 2008; 191(2):396-401. |
| Noguchi Y, Nagata-Kobayashi S, Stahl JE, Wong JB. A meta-analytic comparison of echocardiographic stressors *The International Journal of Cardiovascular Imaging* 2005; 21(2-3):189-207. |
| Numans ME, Lau J, de Wit NJ, Bonis PA. Short-term treatment with proton-pump inhibitors as a test for gastroesophageal reflux disease: a meta-analysis of diagnostic test characteristics. *Annals of Internal Medicine* 2004; 140(7):518-527. |
| Ogilvie GS, Patrick DM, Schulzer M, Sellors JW, Petric M, Chambers K et al. Diagnostic accuracy of self collected vaginal specimens for human papillomavirus compared to clinician collected human papillomavirus specimens: a meta-analysis. *Sexually Transmitted Infections* 2005; 81(3):207-212. |
| Ola B, Afnan M, Papaioannou S, Sharif K, Bjorndahl L, Coomarasamy A. Accuracy of sperm - Cervical mucus penetration tests in evaluating sperm motility in semen: A systematic quantitative review. *Human Reproduction* 2003; 18(5):1037-1046. |
| Owens DK, Holodniy M, Garber AM, Scott J, Sonnad S, Moses L et al. Polymerase chain reaction for the diagnosis of HIV infection in adults. A meta-analysis with recommendations for clinical practice and study design. *Annals of Internal Medicine* 1996; 124(9):803-815. |
| Pai M, Flores LL, Hubbard A, Riley LW, Colford JM, Jr. Nucleic acid amplification tests in the diagnosis of tuberculous pleuritis: a systematic review and meta-analysis. *BMC Infectious Diseases* 2004; 4:6. |
| Pai M, Flores LL, Pai N, Hubbard A, Riley LW, Colford JM, Jr. Diagnostic accuracy of nucleic acid amplification tests for tuberculous meningitis: a systematic review and meta-analysis.*The Lancet Infectious Diseases* 2003; 3(10):633-643. |
| Pai M, Kalantri S, Pascopella L, Riley LW, Reingold AL. Bacteriophage-based assays for the rapid detection of rifampicin resistance in Mycobacterium tuberculosis: a meta-analysis. *Journal of Infection* 2005; 51(3):175-187. |
| Pai NP, Tulsky JP, Cohan D, Colford JM, Jr., Reingold AL. Rapid point-of-care HIV testing in pregnant women: a systematic review and meta-analysis. *Tropical Medicine & International Health* 2007; 12(2):162-173. |
| Pakos EE, Fotopoulos AD, Ioannidis JP. 18F-FDG PET for evaluation of bone marrow infiltration in staging of lymphoma: a meta-analysis. *Journal of Nuclear Medicine* 2005; 46(6):958-963. |
| Pakos EE, Koumoulis HD, Fotopoulos AD, Ioannidis JP. Osteomyelitis: antigranulocyte scintigraphy with 99mTC radiolabeled monoclonal antibodies for diagnosis-- meta-analysis. *Radiology* 2007 ^a^; 245(3):732-741. |
| Pakos EE, Trikalinos TA, Fotopoulos AD, Ioannidis JP. Prosthesis infection: diagnosis after total joint arthroplasty with antigranulocyte scintigraphy with 99mTc-labeled monoclonal antibodies--a meta-analysis. *Radiology* 2007 ^b^; 242(1):101-108. |
| Patwardhan MB, McCrory DC, Matchar DB, Samsa GP, Rutschmann OT. Alzheimer disease: operating characteristics of PET--a meta-analysis. *Radiology* 2004; 231(1):73-80. |
| Peters NH, Borel Rinkes IH, Zuithoff NP, Mali WP, Moons KG, Peeters PH et al. Meta-analysis of MR imaging in the diagnosis of breast lesions. *Radiology* 2008; 246(1):116-124. |
| Pfeiffer CD, Fine JP, Safdar N. Diagnosis of invasive aspergillosis using a galactomannan assay: a meta-analysis. *Clinical Infectious Diseases* 2006; 42(10):1417-1427. |
| Pirozzo S, Papinczak T, Glasziou P. Whispered voice test for screening for hearing impairment in adults and children: a systematic review. *BMJ 2003; 327:967-971* |
| Price CP, Newall RG, Boyd JC. Use of protein:creatinine ratio measurements on random urine samples for prediction of significant proteinuria: a systematic review. *Clinical Chemistry* 2005; 51(9):1577-1586. |
| Puli SR, Batapati Krishna RJ, Bechtold ML, Antillon MR, Ibdah JA, Puli SR et al. How good is endoscopic ultrasound for TNM staging of gastric cancers? A meta-analysis and systematic review. *World Journal of Gastroenterology* 2008^a^; 14(25):4011-4019. |
| Puli SR, Batapati Krishna RJ, Bechtold ML, Ibdah JA, Antillon D, Singh S et al. Endoscopic ultrasound: it's accuracy in evaluating mediastinal lymphadenopathy? A meta-analysis and systematic review. *World Journal of Gastroenterology* 2008 ^b^; 14(19):3028-3037. |
| Puli SR, Reddy JB, Bechtold ML, Antillon D, Ibdah JA, Antillon MR et al. Staging accuracy of esophageal cancer by endoscopic ultrasound: a meta-analysis and systematic review. *World Journal of Gastroenterology* 2008^c^; 14(10):1479-1490. |
| Puli SR, Reddy JB, Bechtold ML, Antillon MR, Ibdah JA, Puli SR et al. Accuracy of endoscopic ultrasound in the diagnosis of distal and celiac axis lymph node metastasis in esophageal cancer: a meta-analysis and systematic review. *Digestive Diseases & Sciences* 2008^d^; 53(9):2405-2414. |
| Puli SR, Singh S, Hagedorn CH, Reddy J, Olyaee M. Diagnostic accuracy of EUS for vascular invasion in pancreatic and periampullary cancers: a meta-analysis and systematic review. *Gastrointestinal Endoscopy* 2007; 65(6):788-797. |
| Purkayastha S, Athanasiou T, Tekkis PP, Constantinides V, Teare J, Darzi AW. Magnetic resonance colonography vs computed tomography colonography for the diagnosis of colorectal cancer: an indirect comparison. *Colorectal Disease* 2007 ^a^; 9(2):100-111. |
| Purkayastha S, Chow A, Athanasiou T, Cambaroudis A, Panesar S, Kinross J et al. Does serum procalcitonin have a role in evaluating the severity of acute pancreatitis? A question revisited. *World Journal of Surgery* 2006; 30(9):1713-1721. |
| Purkayastha S, Tekkis PP, Athanasiou T, Aziz O, Negus R, Gedroyc W et al. Magnetic resonance colonography versus colonoscopy as a diagnostic investigation for colorectal cancer: a meta-analysis. *Clinical Radiology* 2005; 60(9):980-989. |
| Purkayastha S, Tekkis PP, Athanasiou T, Tilney HS, Darzi AW, Heriot AG. Diagnostic precision of magnetic resonance imaging for preoperative prediction of the circumferential margin involvement in patients with rectal cancer. *Colorectal Disease* 2007 ^b^; 9(5):402-411. |
| Reese GE, Constantinides VA, Simillis C, Darzi AW, Orchard TR, Fazio VW et al. Diagnostic precision of anti-Saccharomyces cerevisiae antibodies and perinuclear antineutrophil cytoplasmic antibodies in inflammatory bowel disease. *American Journal of Gastroenterology* 2006; 101(10):2410-2422. |
| Roddam AW, Duffy MJ, Hamdy FC, Ward AM, Patnick J, Price CP et al. Use of prostate-specific antigen (PSA) isoforms for the detection of prostate cancer in men with a PSA level of 2-10 ng/ml: systematic review and meta-analysis. *European Urology* 2005; 48(3):386-399. |
| Rodgers M, Nixon J, Hempel S, Aho T, Kelly J. Diagnostic tests and algorithms used in the investigation of haematuria: systematic reviews and economic evaluation. *Health Technology Assessment* 10(18). |
| Ross SD, Sheinhait IA, Harrison KJ, Kvasz M, Connelly JE, Shea SA et al. Systematic review and meta-analysis of the literature regarding the diagnosis of sleep apnea. *Sleep* 2000; 23(4):519-532. |
| Roy PM, Colombet I, Durieux P, Chatellier G, Sors H, Meyer G. Systematic review and meta-analysis of strategies for the diagnosis of suspected pulmonary embolism. *BMJ* 2005; 331(7511):259-263. |
| Safdar N, Fine JP, Maki DG. Meta-analysis: methods for diagnosing intravascular device-related bloodstream infection. *Annals of Internal Medicine* 2005; 142(6):451-466. |
| Samson DJ, Flamm CR, Pisano ED, Aronson N. Should FDG PET be used to decide whether a patient with an abnormal mammogram or breast finding at physical examination should undergo biopsy? *Academic Radiology* 2002; 9(7):773-783. |
| Sarmiento OL, Weigle KA, Alexander J, Weber DJ, Miller WC. Assessment by meta-analysis of PCR for diagnosis of smear-negative pulmonary tuberculosis. *Journal of Clinical Microbiology* 2003; 41(7):3233-3240. |
| Sauerland S, Bouillon B, Rixen D, Raum MR, Koy T, Neugebauer EA. The reliability of clinical examination in detecting pelvic fractures in blunt trauma patients: a meta-analysis. *Archives of Orthopaedic & Trauma Surgery* 2004; 124(2):123-128. |
| Scholten RJ, Deville WL, Opstelten W, Bijl D, van der Plas CG, Bouter LM. The accuracy of physical diagnostic tests for assessing meniscal lesions of the knee: a meta-analysis.*Journal of Family Practice* 2001; 50(11):938-944. |
| Scholten RJ, Opstelten W, van der Plas CG, Bijl D, Deville WL, Bouter LM. Accuracy of physical diagnostic tests for assessing ruptures of the anterior cruciate ligament: a meta-analysis. *Journal of Family Practice* 2003; 52(9):689-694. |
| Schreiber G, McCrory DC. Performance characteristics of different modalities for diagnosis of suspected lung cancer: summary of published evidence. *Chest* 2003; 123(1 Supplement):115S-128S. |
| Selman TJ, Luesley DM, Acheson N, Khan KS, Mann CH. A systematic review of the accuracy of diagnostic tests for inguinal lymph node status in vulvar cancer. *Gynecologic Oncology* 2005; 99(1):206-214. |
| Selman TJ, Mann C, Zamora J, Appleyard TL, Khan K, Selman TJ et al. Diagnostic accuracy of tests for lymph node status in primary cervical cancer: a systematic review and meta-analysis. *CMAJ Canadian Medical Association Journal* 2008^a^; 178(7):855-862. |
| Selman TJ, Mann CH, Zamora J, Khan KS, Selman TJ, Mann CH et al. A systematic review of tests for lymph node status in primary endometrial cancer. *BMC Women's Health* 2008^b^; 8:8. |
| Shafiq N, Malhotra S, Bhasin DK, Rana S, Siddhu S, Pandhi P. Estimating the diagnostic accuracy of procalcitonin as a marker of the severity of acute pancreatitis: a meta-analytic approach. *Journal of the Pancreas* 2005; 6(3):231-237. |
| Shaheen AA, Myers RP, Shaheen AA, Myers RP. Systematic review and meta-analysis of the diagnostic accuracy of fibrosis marker panels in patients with HIV/hepatitis C coinfection. *HIV Clinical Trials* 2008; 9(1):43-51. |
| Shaheen AA, Myers RP. Diagnostic accuracy of the aspartate aminotransferase-to-platelet ratio index for the prediction of hepatitis C-related fibrosis: a systematic review. *Hepatology* 2007 ^a^; 46(3):912-921. |
| Shaheen AA, Wan AF, Myers RP. FibroTest and FibroScan for the prediction of hepatitis C-related fibrosis: a systematic review of diagnostic test accuracy. *American Journal of Gastroenterology* 2007 ^b^; 102(11):2589-2600. |
| Shi HZ, Liang QL, Jiang J, Qin XJ, Yang HB, Shi HZ et al. Diagnostic value of carcinoembryonic antigen in malignant pleural effusion: a meta-analysis. *Respirology* 2008; 13(4):518-527. |
| Shie P, Cardarelli R, Brandon D, Erdman W, Abdulrahim N, Shie P et al. Meta-analysis: comparison of F-18 Fluorodeoxyglucose-positron emission tomography and bone scintigraphy in the detection of bone metastases in patients with breast cancer. *Clinical Nuclear Medicine* 2008; 33(2):97-101. |
| Shiga T, Wajima Z, Apfel CC, Inoue T, Ohe Y. Diagnostic accuracy of transesophageal echocardiography, helical computed tomography, and magnetic resonance imaging for suspected thoracic aortic dissection: systematic review and meta-analysis. *Archives of Internal Medicine* 2006; 166(13):1350-1356. |
| Song JM, Kim CB, Chung HC, Kane RL. Prostate-specific antigen, digital rectal examination and transrectal ultrasonography: a meta-analysis for this diagnostic triad of prostate cancer in symptomatic korean men. *Yonsei Medical Journal* 2005; 46(3):414-424. |
| Sosna J, Sella T, Sy O, Lavin PT, Eliahou R, Fraifeld S et al. Critical analysis of the performance of double-contrast barium enema for detecting colorectal polyps > or = 6 mm in the era of CT colonography. American Journal of Roentgenology. 2008; 190(2):374-385. |
| Sotiriadis A, Makrydimas G, Ioannidis JP. Diagnostic performance of intracardiac echogenic foci for Down syndrome: a meta-analysis. *Obstetrics & Gynecology* 2003; 101(5:Pt 1):1009-1016. |
| Speight PM, Palmer S, Moles DR, Downer MC, Smith DH. The cost effectiveness of screening for oral cancer in primary care.  *Health Technology Assessment* 10(14). |
| St John A, Boyd JC, Lowes AJ, Price CP. The use of urinary dipstick tests to exclude urinary tract infection: a systematic review of the literature. *American Journal of Clinical Pathology* 2006; 126(3):428-436. |
| Stein PD, Beemath A, Kayali F, Skaf E, Sanchez J, Olson RE. Multidetector computed tomography for the diagnosis of coronary artery disease: A systematic review. *American Journal of Medicine* 2006; 119(3):203-216. |
| Stein PD, Hull RD, Patel KC, Olson RE, Ghali WA, Brant R et al. D-dimer for the exclusion of acute venous thrombosis and pulmonary embolism: a systematic review. *Annals of Internal Medicine* 2004; 140(8):589-602. |
| Steingart KR, Henry M, Laal S, Hopewell PC, Ramsay A, Menzies D et al. A systematic review of commercial serological antibody detection tests for the diagnosis of extrapulmonary tuberculosis. *Postgraduate Medical Journal* 2007 ^a^; 83(985):705-712. |
| Steingart KR, Henry M, Laal S, Hopewell PC, Ramsay A, Menzies D et al. Commercial serological antibody detection tests for the diagnosis of pulmonary tuberculosis: a systematic review. *PLoS Medicine / Public Library of Science* 2007 ^b^; 4(6):e202. |
| Steingart KR, Ng V, Henry M, Hopewell PC, Ramsay A, Cunningham J et al. Sputum processing methods to improve the sensitivity of smear microscopy for tuberculosis: a systematic review.*The Lancet Infectious Diseases* 2006; 6(10):664-674. |
| Stengel D, Bauwens K, Rademacher G, Mutze S, Ekkernkamp A. Association between compliance with methodological standards of diagnostic research and reported test accuracy: meta-analysis of focused assessment of US for trauma. *Radiology* 2005; 236(1):102-111. |
| Stengel D, Bauwens K, Sehouli J, Porzsolt F, Rademacher G, Mutze S et al. Systematic review and meta-analysis of emergency ultrasonography for blunt abdominal trauma. *British Journal of Surgery* 2001; 88(7):901-912. |
| Takata GS, Chan LS, Morphew T, Mangione-Smith R, Morton SC, Shekelle P. Evidence assessment of the accuracy of methods of diagnosing middle ear effusion in children with otitis media with effusion. *Pediatrics* 2003; 112(6:Pt 1):1379-1387. |
| Tang BM, Eslick GD, Craig JC, McLean AS. Accuracy of procalcitonin for sepsis diagnosis in critically ill patients: systematic review and meta-analysis. *The Lancet Infectious Diseases* 2007; 7(3):210-217. |
| Terasawa T, Blackmore CC, Bent S, Kohlwes RJ. Systematic review: computed tomography and ultrasonography to detect acute appendicitis in adults and adolescents. *Annals of Internal Medicine* 2004; 141(7):537-546. |
| Termaat MF, Raijmakers PG, Scholten HJ, Bakker FC, Patka P, Haarman HJ. The accuracy of diagnostic imaging for the assessment of chronic osteomyelitis: a systematic review and meta-analysis. *Journal of Bone & Joint Surgery - American Volume* 2005; 87(11):2464-2471. |
| Tew K, Irwig L, Matthews A, Crowe P, Macaskill P. Meta-analysis of sentinel node imprint cytology in breast cancer. *British Journal of Surgery* 2005; 92(9):1068-1080. |
| Trochez-Martinez RD, Smith P, Lamont RF. Use of C-reactive protein as a predictor of chorioamnionitis in preterm prelabour rupture of membranes: a systematic review. *BJOG: An International Journal of Obstetrics & Gynaecology* 2007; 114(7):796-801. |
| Trowbridge RL, Rutkowski NK, Shojania KG. The rational clinical examination. Does this patient have acute cholecystitis? *JAMA.* 2003; 289(1):80-86. |
| Tse F, Liu L, Barkun AN, Armstrong D, Moayyedi P, Tse F et al. EUS: a meta-analysis of test performance in suspected choledocholithiasis. *Gastrointestinal Endoscopy* 2008; 67(2):235-244. |
| Tuon FF, Litvoc MN, Lopes MI. Adenosine deaminase and tuberculous pericarditis--a systematic review with meta-analysis. *Acta Tropica* 2006; 99(1):67-74. |
| Tuon FF. A systematic literature review on the diagnosis of invasive aspergillosis using polymerase chain reaction (PCR) from bronchoalveolar lavage clinical samples. *Revista Iberoamericana de Micologia* 2007; 24(2):89-94. |
| Vakil N, Moayyedi P, Fennerty MB, Talley NJ. Limited value of alarm features in the diagnosis of upper gastrointestinal malignancy: systematic review and meta-analysis. *Gastroenterology* 659; 131(2):390-401. |
| van Dongen H, de Kroon CD, Jacobi CE, Trimbos JB, Jansen FW. Diagnostic hysteroscopy in abnormal uterine bleeding: a systematic review and meta-analysis. *BJOG: An International Journal of Obstetrics & Gynaecology* 2007; 114(6):664-675. |
| van Randen A, Bipat S, Zwinderman AH, Ubbink DT, Stoker J, Boermeester MA et al. Acute appendicitis: meta-analysis of diagnostic performance of CT and graded compression US related to prevalence of disease. *Radiology* 2008; 249(1):97-106. |
| van Westreenen HL, Westerterp M, Bossuyt PM, Pruim J, Sloof GW, van Lanschot JJ et al. Systematic review of the staging performance of 18F-fluorodeoxyglucose positron emission tomography in esophageal cancer. *Journal of Clinical Oncology* 2004; 22(18):3805-3812. |
| Van Zaane B, Zuithoff NP, Reitsma JB, Bax L, Nierich AP, Moons KG et al. Meta-analysis of the diagnostic accuracy of transesophageal echocardiography for assessment of atherosclerosis in the ascending aorta in patients undergoing cardiac surgery. *Acta Anaesthesiologica Scandinavica* 2008; 52(9):1179-1187. |
| Vanezis AP, Bhopal R, Vanezis AP, Bhopal R. Validity of electrocardiographic classification of left ventricular hypertrophy across adult ethnic groups with echocardiography as a standard. *Journal of Electrocardiology* 2008; 41(5):404-412. |
| Vanhoenacker PK, Decramer I, Bladt O, Sarno G, Bevernage C, Wijns W. Detection of non-ST-elevation myocardial infarction and unstable angina in the acute setting: meta-analysis of diagnostic performance of multi-detector computed tomographic angiography. *BMC Cardiovascular Disorders* 2007; 7:39. |
| Vasbinder GB, Nelemans PJ, Kessels AG, Kroon AA, de Leeuw PW, van Engelshoven JM. Diagnostic tests for renal artery stenosis in patients suspected of having renovascular hypertension: a meta-analysis. *Annals of Internal Medicine* 2001; 135(6):401-411. |
| Vestergaard ME, Macaskill P, Holt PE, Menzies SW, Vestergaard ME, Macaskill P et al. Dermoscopy compared with naked eye examination for the diagnosis of primary melanoma: a meta-analysis of studies performed in a clinical setting. *British Journal of Dermatology* 2008; 159(3):669-676. |
| Virgili G, Menchini F, Dimastrogiovanni AF, Rapizzi E, Menchini U, Bandello F et al. Optical coherence tomography versus stereoscopic fundus photography or biomicroscopy for diagnosing diabetic macular edema: a systematic review. *Investigative Ophthalmology & Visual Science* 2007; 48(11):4963-4973. |
| Vlaar AM, van Kroonenburgh MJ, Kessels AG, Weber WE. Meta-analysis of the literature on diagnostic accuracy of SPECT in parkinsonian syndromes. *BMC Neurology* 2007; 7:27. |
| von Roon AC, Karamountzos L, Purkayastha S, Reese GE, Darzi AW, Teare JP et al. Diagnostic precision of fecal calprotectin for inflammatory bowel disease and colorectal malignancy. *American Journal of Gastroenterology* 2007; 102(4):803-813. |
| Vroomen PC, de Krom MC, Knottnerus JA. Diagnostic value of history and physical examination in patients suspected of sciatica due to disc herniation: a systematic review. *Journal of Neurology* 1999; 246(10):899-906. |
| Wang P, Guo YM, Liu M, Qiang YQ, Guo XJ, Zhang YL et al. A meta-analysis of the accuracy of prostate cancer studies which use magnetic resonance spectroscopy as a diagnostic tool. *Korean Journal of Radiology* 2008; 9(5):432-438. |
| Wang WH, Huang JQ, Zheng GF, Wong WM, Lam SK, Karlberg J et al. Is proton pump inhibitor testing an effective approach to diagnose gastroesophageal reflux disease in patients with noncardiac chest pain?: a meta-analysis. *Archives of Internal Medicine* 2005; 165(11):1222-1228. |
| Wang Y, Sun G, Pan JG, Guo ZJ, Li T. Performance of tPSA and f/tPSA for prostate cancer in Chinese. A systematic review and meta-analysis. *Prostate Cancer & Prostatic Diseases* 2006; 9(4):374-378. |
| Wardlaw JM, Chappell FM, Stevenson M, De NE, Thomas S, Gillard J et al. Accurate, practical and cost-effective assessment of carotid stenosis in the UK. *Health Technology Assessment* 2006; 10(30):1-128. |
| White PM, Wardlaw JM, Easton V. Can noninvasive imaging accurately depict intracranial aneurysms? A systematic review.*Radiology* 2000; 217(2):361-370. |
| Whiting P, Harbord R, Main C, Decks JJ, Filippini G, Egger M et al. Accuracy of magnetic resonance imaging for the diagnosis of multiple sclerosis: Systematic review. *British Medical Journal* 2006^a^; 332(7546):875-878. |
| Whiting P, Westwood M, Bojke L, Palmer S, Richardson G, Cooper J et al. Clinical effectiveness and cost-effectiveness of tests for the diagnosis and investigtion of urinary tract infection in children: A systematic review and economic model. *Health Technology Assessment* 2006^b^; 10(36):1-154. |
| Whitsel EA, Boyko EJ, Siscovick DS. Reassessing the role of QTc in the diagnosis of autonomic failure among patients with diabetes: a meta-analysis. *Diabetes Care* 2000; 23(2):241-247. |
| Will O, Purkayastha S, Chan C, Athanasiou T, Darzi AW, Gedroyc W et al. Diagnostic precision of nanoparticle-enhanced MRI for lymph-node metastases: a meta-analysis. *Lancet Oncology* 2006; 7(1):52-60. |
| Williams GJ, Macaskill P, Chan SF, Karplus TE, Yung W, Hodson EM et al. Comparative accuracy of renal duplex sonographic parameters in the diagnosis of renal artery stenosis: paired and unpaired analysis. *American Journal of Roentgenology* 2007; 188(3):798-811. |
| Wittkampf KA, Naeije L, Schene AH, Huyser J, van Weert HC. Diagnostic accuracy of the mood module of the Patient Health Questionnaire: a systematic review. *General Hospital Psychiatry* 2007; 29(5):388-395. |
| Worster A, Balion CM, Hill SA, Santaguida P, Ismaila A, McKelvie R et al. Diagnostic accuracy of BNP and NT-proBNP in patients presenting to acute care settings with dyspnea: a systematic review. *Clinical Biochemistry* 2008; 41(4-5):250-259. |
| Worster A, Preyra I, Weaver B, Haines T. The accuracy of noncontrast helical computed tomography versus intravenous pyelography in the diagnosis of suspected acute urolithiasis: a meta-analysis. *Annals of Emergency Medicine* 2002; 40(3):280-286. |
| Wykes CB, Clark TJ, Khan KS. Accuracy of laparoscopy in the diagnosis of endometriosis: A systematic quantitative review.  *BJOG: An International Journal of Obstetrics and Gynaecology* 2004; 111(11):1204-1212. |
