# Supplementary material for: The assessment of the quality of reporting of meta-analyses in diagnostic research: a systematic review
Source: BMC Med Res Methodol. 2011 Dec 9;11:163. doi: 10.1186/1471-2288-11-163 (PMC3258221; doi:10.1186/1471-2288-11-163)
Supplement: Additional file 3 — Appendix 3 - PRISMA results for individual reviews. [file 1471-2288-11-163-S3.DOC]

**Appendix 3. PRISMA results for individual reviews (PRISMA items 1-27)**

| Studies | | **1** | **2** | **3** | **4** | **5** | **6** | **7** | **8** | **9** | **10** | **11** | **12** | **13** | **14** | **15** | **16** | **17** | **18** | **19** | **20** | **21** | **22** | **23** | **24** | **25** | **26** | **27** |
| --- | --- | --- | --- | --- | --- | --- | --- | --- | --- | --- | --- | --- | --- | --- | --- | --- | --- | --- | --- | --- | --- | --- | --- | --- | --- | --- | --- | --- |
| Abdulla | 2007 | 1 | P | 1 | P | 0 | 1 | 1 | P | 0 | P | 1 | 0 | 1 | 1 | P | P | P | 1 | 0 | 0 | 1 | 1 | 1 | 1 | 1 | 1 | 1 |
| Abubakar | 2007 | 1 | 1 | 1 | 1 | P | 1 | 1 | 1 | 1 | 1 | 1 | 0 | 1 | 1 | 1 | 0 | 1 | 1 | 0 | 1 | 1 | 1 | 0 | 1 | 1 | 1 | 1 |
| Akcil | 2008 | 1 | P | 1 | P | 0 | 1 | 1 | P | P | 1 | P | P | 1 | 1 | 1 | 1 | P | P | 0 | 1 | 1 | 1 | 1 | 1 | 1 | 1 | 0 |
| Arbyn | 2008 | 1 | 1 | 1 | P | 0 | 1 | 1 | P | 1 | 1 | 1 | P | 1 | 1 | 1 | 1 | P | 1 | 1 | 0 | 1 | 1 | 1 | 1 | P | 1 | 0 |
| Arbyn | 2004 | 1 | P | 1 | 1 | 0 | 1 | 1 | 1 | 0 | 0 | 1 | P | 1 | 1 | 1 | 0 | 1 | 1 | 0 | 1 | 1 | 1 | 1 | 1 | P | 1 | 0 |
| Atieh | 2008 | 1 | P | 1 | P | 0 | 1 | 1 | P | 0 | 0 | 1 | 0 | 1 | 1 | 1 | 0 | 1 | 1 | 0 | P | 1 | 1 | 0 | 1 | 1 | 1 | 1 |
| Bafounta | 2001 | 1 | 1 | 1 | P | 0 | 1 | 1 | P | 1 | 0 | 1 | 1 | 1 | 1 | 0 | 0 | 0 | 1 | 0 | P | 1 | 0 | 0 | 1 | 1 | 1 | 1 |
| Bafounta | 2004 | 1 | P | 1 | 1 | 0 | P | 1 | P | 0 | 1 | 1 | 0 | 1 | 1 | 0 | 0 | P | P | 0 | 1 | 1 | 0 | 0 | P | 1 | P | 0 |
| Bagai | 2006 | P | 1 | 1 | P | 0 | 1 | 1 | P | 1 | 1 | P | 1 | 1 | 1 | P | 0 | P | 1 | 1 | 1 | 1 | 0 | 0 | 1 | 0 | 1 | 1 |
| Bakis | 2004 | 1 | 1 | 1 | P | 0 | 1 | 1 | P | 1 | P | P | 1 | 1 | 1 | P | 0 | P | P | 1 | 1 | 1 | 0 | 0 | 1 | 1 | 1 | 0 |
| Barnes | 2002 | 1 | P | 1 | P | 0 | P | 1 | P | 0 | P | P | 0 | 1 | 1 | 0 | 0 | P | P | 0 | P | 1 | 0 | 0 | 1 | 1 | 1 | 0 |
| Bastian | 1998 | 1 | 1 | 1 | P | 0 | 1 | 1 | P | 0 | 1 | 0 | 0 | 1 | 1 | P | 0 | P | 1 | 0 | P | 1 | 0 | 0 | 1 | P | 1 | 0 |
|  |  | **1** | **2** | **3** | **4** | **5** | **6** | **7** | **8** | **9** | **10** | **11** | **12** | **13** | **14** | **15** | **16** | **17** | **18** | **19** | **20** | **21** | **22** | **23** | **24** | **25** | **26** | **27** |
| Benjaminse | 2006 | 1 | 1 | 1 | P | 0 | 1 | 1 | 1 | 1 | 1 | 0 | 1 | 1 | 1 | 1 | 1 | 1 | P | 1 | 1 | 1 | P | P | 1 | 1 | 1 | 1 |
| Berner | 2007 | 1 | 1 | 1 | 1 | P | 1 | 1 | P | 1 | 1 | 0 | 1 | 1 | 1 | 1 | P | 1 | 1 | 1 | 1 | 1 | 1 | 0 | 1 | 1 | 1 | 0 |
| Berry | 1999 | 1 | 0 | 1 | 1 | P | 1 | 1 | 1 | 1 | 1 | 1 | P | 1 | 1 | 0 | 1 | P | 1 | P | 1 | 1 | 0 | 1 | 1 | 0 | 1 | 1 |
| Berry | 2002 | 1 | 0 | 1 | 1 | P | 1 | 1 | 1 | P | 1 | 1 | 1 | 1 | 1 | P | 1 | P | P | P | 1 | 1 | 0 | 1 | 1 | 0 | 1 | 1 |
| Bipat | 2004 | 1 | P | 1 | P | 0 | 1 | 1 | P | 1 | 1 | 1 | 1 | 1 | 1 | 1 | 1 | P | P | 1 | 0 | 1 | P | 1 | 1 | 1 | 1 | 0 |
| Bipat | 2003 | 1 | 1 | 1 | P | 0 | 1 | 1 | P | 0 | 1 | 1 | 1 | 1 | 1 | 0 | 1 | P | P | 1 | 0 | 1 | 0 | 1 | 1 | 1 | 1 | 0 |
| Bipat | 2005 | 1 | P | 1 | P | 0 | 1 | 1 | P | 1 | 1 | 1 | 1 | 1 | 1 | P | 1 | 0 | P | 1 | 1 | 1 | P | 1 | 1 | P | 1 | 0 |
| Blacksell | 2006 | 1 | 0 | 1 | 1 | 0 | 1 | 1 | P | P | 1 | 0 | P | 1 | 1 | 1 | 1 | 1 | 1 | P | 1 | 1 | 1 | 1 | 1 | P | 1 | 1 |
| Brealey | 2005 | 1 | P | 1 | 1 | 0 | 1 | 1 | P | P | 1 | P | P | 1 | 1 | 1 | 1 | 1 | 1 | P | 1 | 1 | 1 | 1 | 1 | 0 | 1 | 0 |
| Brown | 2003 | 1 | 1 | 1 | 1 | 0 | 1 | 1 | P | 1 | 1 | 1 | 1 | 1 | 1 | 0 | 0 | 1 | 1 | P | 1 | 1 | 0 | 0 | 1 | 0 | 1 | 0 |
| Brown | 2002 | 1 | P | 1 | 1 | 0 | P | 1 | P | 1 | 1 | 0 | P | 1 | P | 0 | 0 | 1 | 1 | P | 1 | 1 | 0 | 0 | 1 | 1 | 1 | 0 |
| Bruyninckx | 2008 | 1 | P | 1 | 1 | 0 | 1 | 1 | 1 | 1 | 1 | 1 | 1 | 1 | 1 | 1 | 0 | 1 | 1 | 1 | 1 | 1 | 0 | 0 | 1 | 1 | 1 | 0 |
| Burr | 2007 | 1 | P | 1 | 1 | P | 1 | 1 | 1 | 1 | 1 | 1 | 0 | 1 | 1 | 0 | 0 | 0 | 1 | 0 | 1 | 1 | 0 | 0 | 1 | 1 | 1 | 1 |
|  |  | **1** | **2** | **3** | **4** | **5** | **6** | **7** | **8** | **9** | **10** | **11** | **12** | **13** | **14** | **15** | **16** | **17** | **18** | **19** | **20** | **21** | **22** | **23** | **24** | **25** | **26** | **27** |
| Campens | 1997 | 1 | P | 1 | P | 0 | 1 | 1 | P | 0 | 0 | 0 | P | 1 | 1 | 0 | 0 | 0 | 1 | 0 | P | 1 | 1 | 0 | 1 | 1 | 1 | 0 |
| Castilla-Rilo | 2007 | 1 | 1 | 1 | 1 | 0 | 1 | 1 | P | 0 | 0 | P | 0 | 1 | 1 | 0 | 0 | 1 | 1 | 0 | 1 | 1 | 0 | 0 | 1 | 1 | 1 | 1 |
| Cavallazzi | 2008 | 1 | P | 1 | P | 0 | 1 | 1 | P | 1 | 1 | 1 | P | 1 | 1 | 0 | 0 | 1 | P | 0 | P | 1 | P | 0 | 1 | 0 | 1 | 0 |
| Cepoiu | 2008 | 1 | P | 1 | 1 | 0 | 1 | 1 | P | 1 | 0 | 1 | P | 1 | 1 | 1 | 1 | 1 | 1 | 1 | P | 1 | 0 | 1 | 1 | 0 | 1 | 1 |
| Chalco | 2005 | 1 | P | 1 | 1 | 0 | 1 | 1 | 1 | 1 | 0 | 0 | 1 | 1 | 1 | 1 | 1 | P | 1 | 0 | 1 | 1 | 1 | 1 | 1 | P | 1 | 1 |
| Chen | 2001 | 1 | 1 | 1 | P | 0 | 1 | 1 | P | P | 1 | 1 | P | 1 | 1 | 0 | 0 | P | P | 0 | P | 1 | 0 | 0 | 1 | 1 | P | 1 |
| Chua | 2008 | P | P | 1 | P | 0 | 1 | 1 | P | 0 | 0 | P | P | 1 | 1 | 1 | 0 | 0 | P | P | 1 | 1 | 1 | 0 | 1 | 1 | 1 | 0 |
| Clark | 2001 | 0 | 1 | 1 | 1 | 0 | 1 | 1 | P | 1 | 0 | P | 1 | 1 | 1 | 1 | 1 | 1 | P | 1 | 1 | 1 | 1 | 1 | P | P | 1 | 1 |
| Clark | 2002 | 1 | 1 | 1 | 1 | 0 | 1 | 1 | P | 1 | 0 | 1 | 1 | 1 | 1 | 1 | 1 | 1 | 1 | P | 0 | 1 | 1 | 1 | 1 | 0 | 1 | 1 |
| Clark | 2000 | 1 | 1 | 1 | P | 0 | P | 1 | P | 0 | 0 | P | 0 | 1 | 1 | P | 0 | 0 | P | P | 0 | 1 | 0 | 0 | 1 | 0 | 1 | 0 |
| Colin | 2001 | 1 | P | 1 | P | P | P | 1 | P | 0 | 0 | 1 | P | 1 | 1 | 1 | 0 | P | P | P | P | 1 | 1 | 0 | 1 | P | 1 | 1 |
| Dales | 1990 | 1 | P | 1 | P | 0 | P | 1 | P | 0 | 0 | 1 | 1 | 1 | 1 | 1 | 1 | P | 1 | 1 | P | 1 | P | 1 | 1 | 1 | 1 | 0 |
| de Bondt | 2007 | 1 | 1 | 1 | 1 | 0 | 1 | 1 | P | P | 1 | 1 | 0 | 1 | 1 | P | 0 | P | P | 0 | 1 | 1 | 0 | 0 | 1 | P | 1 | 0 |
|  |  | **1** | **2** | **3** | **4** | **5** | **6** | **7** | **8** | **9** | **10** | **11** | **12** | **13** | **14** | **15** | **16** | **17** | **18** | **19** | **20** | **21** | **22** | **23** | **24** | **25** | **26** | **27** |
| de Kroon | 2003 | 1 | P | 1 | 1 | 0 | 1 | 1 | P | 1 | 1 | P | 1 | 1 | 1 | 1 | 0 | 1 | P | 1 | 0 | 1 | 1 | 0 | 1 | 0 | 1 | 0 |
| Debrey | 2008 | 1 | 1 | 1 | P | 0 | 1 | 1 | P | 0 | 0 | P | P | 1 | 1 | 1 | 0 | 1 | 1 | P | 1 | 1 | 1 | 0 | 1 | 1 | 1 | 1 |
| Delgado-Bolton | 2003 | 1 | 1 | 1 | 1 | P | 1 | 1 | 1 | 1 | 1 | P | P | 1 | 1 | 0 | 1 | P | P | 1 | 1 | 1 | 1 | 1 | 1 | 0 | 1 | 1 |
| Deville | 2000 | 1 | 1 | 1 | P | 0 | 1 | 1 | P | 1 | 0 | 1 | 1 | 1 | 1 | 1 | 1 | P | P | 1 | 1 | 1 | 1 | 1 | 1 | 1 | 1 | 0 |
| Deville | 2004 | 1 | 1 | 1 | P | 0 | 1 | 1 | 1 | P | 0 | 1 | 1 | 1 | 1 | 1 | 1 | 1 | 1 | 1 | 0 | 1 | 1 | 1 | 1 | 0 | 1 | 1 |
| Di Fabio | 1996 | 1 | 1 | 1 | P | 0 | 1 | 1 | P | 1 | 0 | P | P | 1 | 1 | P | 1 | 0 | P | 0 | 0 | 1 | P | P | 1 | 1 | 1 | 0 |
| Di Nisio | 2007 | 1 | P | 1 | 1 | 0 | 1 | 1 | P | 1 | 1 | 1 | 1 | 1 | 1 | 1 | 1 | 1 | P | P | 0 | 1 | P | 1 | 1 | 0 | 1 | 1 |
| Dinh | 2008 | 1 | P | 1 | 1 | P | 1 | 1 | P | 0 | 0 | 0 | P | 1 | 1 | 1 | 0 | 1 | P | P | 1 | 1 | 1 | 0 | 1 | 1 | 1 | 1 |
| Dinnes | 2007 | 1 | P | 1 | 1 | P | 1 | 1 | 1 | 1 | 1 | 1 | 1 | 1 | 1 | 1 | 1 | 1 | 1 | 1 | 1 | 1 | 1 | 1 | 1 | 1 | 1 | 1 |
| Dinnes | 2003 | 1 | 1 | 1 | 1 | 1 | 1 | 1 | 1 | 1 | 1 | 1 | 0 | 1 | 1 | 1 | 0 | 1 | 1 | 0 | 1 | 1 | 1 | 0 | 1 | 1 | 1 | 1 |
| Dong | 2008 | 1 | P | 1 | 1 | 0 | 1 | 1 | P | P | 1 | P | 0 | 1 | 1 | 1 | 0 | P | P | 0 | 1 | 1 | 1 | 0 | 1 | P | 1 | 0 |
| Doria | 2006 | 1 | P | 1 | 1 | 0 | 1 | 1 | P | 1 | P | 0 | 0 | 1 | 1 | 1 | 1 | 0 | 1 | P | P | 1 | 1 | 1 | 1 | 1 | 1 | 0 |
| Dubin | 2005 | 1 | P | 1 | P | 0 | 1 | 1 | P | 0 | 1 | 1 | 0 | 1 | 1 | P | 0 | 1 | P | 0 | 1 | 1 | P | 0 | 1 | P | 1 | 0 |
|  |  | **1** | **2** | **3** | **4** | **5** | **6** | **7** | **8** | **9** | **10** | **11** | **12** | **13** | **14** | **15** | **16** | **17** | **18** | **19** | **20** | **21** | **22** | **23** | **24** | **25** | **26** | **27** |
| Ebell | 2004 | 1 | P | P | P | 0 | 1 | 1 | P | 1 | 1 | 0 | 0 | 1 | 1 | 0 | 0 | 0 | 1 | 0 | 0 | 1 | 0 | 0 | 1 | 1 | 1 | 0 |
| Engelbrecht | 2002 | 1 | P | 1 | 1 | 0 | P | 1 | P | 1 | 0 | 1 | P | 1 | 1 | P | 1 | P | 0 | P | 0 | 1 | P | P | 1 | P | 1 | 1 |
| Ewald | 2004 | 1 | 1 | 1 | 1 | 0 | 1 | 1 | P | 0 | 0 | 0 | 0 | 1 | 1 | 1 | 0 | P | 1 | 0 | P | 1 | 1 | 0 | 1 | 0 | 1 | 1 |
| Ewald | 2008 | 1 | P | 1 | 1 | 0 | 1 | 1 | 1 | 0 | 1 | 0 | P | 1 | 1 | 1 | 1 | 1 | 1 | 0 | 1 | 1 | 1 | P | 1 | P | 1 | 0 |
| Fancher | 2004 | 1 | 1 | 1 | 1 | 0 | 1 | 1 | 1 | 1 | 1 | 0 | P | 1 | 1 | 0 | 0 | 1 | 1 | P | 0 | 1 | 0 | 0 | 1 | 1 | 1 | 1 |
| Fischer | 2001 | 1 | P | 1 | P | 0 | 1 | 1 | P | 0 | 0 | 1 | 1 | 1 | 1 | 0 | 0 | P | 0 | P | 0 | 1 | 0 | 0 | 1 | P | 1 | 0 |
| Flores | 2005 | 1 | P | 1 | P | 0 | 1 | 1 | P | P | P | 0 | P | 1 | 1 | P | 1 | 0 | P | P | 0 | 1 | 1 | 1 | 1 | 1 | 1 | 1 |
| Ford | 2008 | 1 | P | 1 | 1 | 0 | 1 | 1 | P | P | 1 | P | P | 1 | 1 | 1 | 1 | 1 | 1 | P | 1 | 1 | P | 1 | 1 | 1 | 1 | 1 |
| Fraquelli | 2005 | 1 | P | 1 | 1 | 0 | 1 | 1 | 1 | 1 | 1 | P | 1 | 1 | 1 | 1 | 1 | P | P | P | 0 | 1 | P | 0 | 1 | 1 | 1 | 1 |
| Friedrich-Rust | 2008 | 1 | P | 1 | P | 0 | 1 | 1 | P | 1 | 1 | 0 | 1 | 1 | 1 | P | 1 | P | P | P | P | 1 | 0 | 1 | 1 | P | 1 | 0 |
| Geifman-Holtzman | 2006 | 1 | P | 1 | 1 | 0 | 1 | 1 | P | 0 | 0 | 1 | 0 | 1 | 1 | 0 | 0 | 1 | P | 0 | P | 1 | 0 | 0 | 1 | 0 | 1 | 0 |
| Gisbert | 2006 | 1 | P | 1 | P | P | 1 | 1 | P | 0 | 1 | 1 | 1 | 1 | 1 | 1 | 1 | 0 | 1 | 1 | 1 | 1 | 1 | 1 | 1 | 0 | 1 | 1 |
| Glas | 2003 | 1 | 1 | 1 | P | 0 | 1 | 1 | P | 1 | 1 | P | P | 1 | 1 | P | 1 | P | P | 1 | 1 | 1 | P | 1 | 1 | 1 | 1 | 0 |
|  |  | **1** | **2** | **3** | **4** | **5** | **6** | **7** | **8** | **9** | **10** | **11** | **12** | **13** | **14** | **15** | **16** | **17** | **18** | **19** | **20** | **21** | **22** | **23** | **24** | **25** | **26** | **27** |
| Goodacre | 2006 | P | P | 1 | 1 | P | P | 1 | 1 | 1 | 0 | 1 | 1 | 1 | 1 | 1 | 1 | P | 1 | 1 | 1 | 1 | P | 1 | 1 | 1 | 1 | 1 |
| Gordon | 2003 | 1 | P | 1 | 1 | 0 | P | 1 | P | 0 | 1 | 0 | 0 | 1 | 1 | 1 | 0 | 1 | P | 0 | 1 | 1 | 1 | 0 | 1 | 0 | 1 | 1 |
| Goto | 2003 | 1 | 1 | 1 | P | 0 | P | 1 | P | 1 | 1 | 1 | 1 | 1 | 1 | P | 1 | P | P | 1 | 1 | 1 | P | 1 | 1 | 1 | 1 | 0 |
| Gould | 2003 | 1 | 1 | 1 | 1 | 0 | 1 | 1 | 1 | 1 | 1 | 1 | P | 1 | 1 | 1 | P | 1 | P | P | 1 | 1 | 1 | P | 1 | 1 | 1 | 1 |
| Gu | 2007 | 1 | P | 1 | 1 | 0 | 1 | 1 | P | 1 | 1 | P | 1 | 1 | 1 | 1 | 1 | P | P | 1 | 1 | 1 | 1 | 1 | 1 | 1 | 1 | 0 |
| Gupta | 2002 | 1 | P | 1 | 1 | 0 | 1 | 1 | P | 1 | 1 | P | 1 | 1 | 1 | 1 | 0 | P | P | P | 1 | 1 | P | 0 | 1 | 0 | 1 | 0 |
| Hallan | 1997 | 1 | P | 1 | P | 0 | 1 | 1 | P | 1 | 0 | 0 | P | 1 | 1 | 0 | P | P | 1 | P | P | 1 | 0 | P | 1 | 1 | 1 | 0 |
| Halligan | 2005 | 1 | 1 | 1 | 1 | 0 | 1 | 1 | P | 1 | 1 | 1 | 1 | 1 | 1 | 1 | 0 | P | P | 0 | 1 | 1 | P | 0 | 1 | 1 | 1 | 0 |
| Hamon | 2008^b^ | 1 | P | 1 | P | 0 | 1 | 1 | P | 0 | 1 | 1 | 1 | 1 | 1 | 1 | P | 1 | 1 | 1 | 1 | 1 | 1 | 0 | 1 | 1 | 1 | 0 |
| Hamon | 2008^a^ | 1 | 1 | 1 | 1 | 0 | 1 | 1 | P | 1 | 1 | 1 | 1 | 1 | 1 | 1 | 0 | 1 | 1 | 1 | 1 | 1 | 1 | 0 | 1 | P | 1 | 0 |
| Hancock | 2007 | 1 | 1 | 1 | 1 | 0 | 1 | 1 | 1 | 1 | P | P | 1 | 1 | 1 | 1 | 0 | 1 | P | 1 | 0 | 1 | P | 0 | 1 | 1 | 1 | 1 |
| Hayashino | 2005 | 1 | 1 | 1 | P | 0 | 1 | 1 | P | 0 | 1 | 1 | P | 1 | 1 | 1 | 1 | 1 | 1 | P | 1 | 1 | 0 | 1 | 1 | 1 | 1 | 1 |
| Hegedus | 2007 | 1 | 1 | 1 | 0 | 0 | 1 | 1 | 1 | 1 | 0 | 0 | 1 | 1 | 1 | 1 | 0 | 1 | P | 0 | 1 | 1 | 1 | 0 | 1 | 1 | 1 | 0 |
|  |  | **1** | **2** | **3** | **4** | **5** | **6** | **7** | **8** | **9** | **10** | **11** | **12** | **13** | **14** | **15** | **16** | **17** | **18** | **19** | **20** | **21** | **22** | **23** | **24** | **25** | **26** | **27** |
| Hegedus | 2008 | 1 | P | 1 | P | 0 | 1 | 1 | 1 | 1 | 0 | P | P | 1 | 1 | 1 | 0 | 1 | P | P | 1 | 1 | 0 | 0 | 1 | 0 | 1 | 1 |
| Heijenbrok-Kal | 2007^a^ | 1 | 1 | 1 | P | 0 | 1 | 1 | 1 | 0 | 0 | 1 | P | 1 | 1 | 1 | 1 | 0 | P | 0 | 1 | 1 | 1 | 1 | 1 | 1 | 1 | 0 |
| Heijenbrok-Kal | 2007 ^b^ | 1 | 1 | 1 | 1 | 0 | 1 | 1 | P | 1 | 1 | 1 | P | 1 | 1 | 1 | 1 | P | 1 | 0 | 1 | 1 | 0 | 1 | 1 | 1 | 1 | 0 |
| Hobby | 2001 | P | P | 1 | P | 0 | 1 | 1 | 1 | 0 | 0 | 0 | P | 1 | 1 | P | 0 | P | P | P | 1 | 1 | P | 0 | 1 | 0 | 1 | 1 |
| Hofman | 2000 | 1 | P | 1 | 1 | 0 | P | 1 | P | 0 | 0 | 1 | P | 1 | 1 | 0 | 1 | P | P | P | P | 1 | 0 | 1 | 1 | P | 1 | 0 |
| Holmes | 2007 | 1 | 1 | 1 | 1 | 0 | 1 | 1 | P | 1 | 1 | 0 | P | 1 | 1 | P | 0 | 0 | 1 | 0 | 0 | 1 | 0 | 0 | 1 | 1 | 1 | 0 |
| Holroyd-Leduc | 2008 | P | 1 | 1 | P | 0 | 1 | 1 | P | 1 | 1 | P | 0 | 1 | 1 | P | 0 | 1 | P | 0 | 1 | 1 | P | 0 | 1 | 1 | 1 | 1 |
| Holty | 2005 | 1 | P | 1 | 1 | 0 | 1 | 1 | 1 | P | 1 | 0 | 1 | 1 | 1 | 1 | 1 | 1 | 1 | P | 1 | 1 | 1 | 1 | 1 | 1 | 1 | 1 |
| Horsthuis | 2008 | 1 | P | 1 | P | 0 | 1 | 1 | 1 | 1 | 1 | 1 | 1 | 1 | 1 | 0 | 0 | 1 | 1 | 1 | 1 | 1 | 0 | 0 | 1 | 1 | 1 | 0 |
| Hovels | 2008 | 1 | P | 1 | 1 | 0 | 1 | 1 | P | 1 | 0 | P | 0 | 1 | 1 | P | 1 | P | 1 | 0 | 1 | 1 | P | P | 1 | 1 | 1 | 0 |
| Huicho | 2002 | 1 | 0 | 1 | 1 | 0 | 1 | 1 | 1 | 1 | 1 | 1 | P | 1 | 1 | P | 1 | P | 1 | P | P | 1 | 0 | 1 | 1 | 1 | 1 | 1 |
| Ioannidis | 2003 | 1 | P | 1 | P | P | P | 1 | P | 0 | 0 | 1 | 0 | 1 | 1 | P | 0 | P | 1 | 0 | P | 1 | 0 | 0 | 1 | 0 | 1 | 1 |
| Jahromi | 2005 | 1 | P | 1 | P | 0 | 1 | 1 | P | 1 | 1 | 1 | 1 | 1 | 1 | 1 | 0 | P | 1 | P | 0 | 1 | 1 | 0 | 1 | 1 | 1 | 0 |
|  |  | **1** | **2** | **3** | **4** | **5** | **6** | **7** | **8** | **9** | **10** | **11** | **12** | **13** | **14** | **15** | **16** | **17** | **18** | **19** | **20** | **21** | **22** | **23** | **24** | **25** | **26** | **27** |
| Jiang | 2007 | 1 | P | 1 | 1 | 0 | 1 | 1 | P | 1 | P | 1 | P | 1 | 1 | 1 | 1 | 0 | P | 1 | P | 1 | 1 | 1 | 1 | 1 | 1 | 1 |
| Jones | 2005 | 1 | P | 1 | 1 | 0 | P | 1 | P | 0 | 1 | 1 | 1 | 1 | 1 | 1 | 1 | 0 | P | 0 | 1 | 1 | P | 1 | 1 | 1 | 1 | 0 |
| Joshi | 2007 | 1 | P | 1 | 1 | 0 | 1 | 1 | 1 | 1 | 1 | 1 | P | 1 | 1 | 1 | 0 | P | P | 1 | 0 | 1 | 0 | 0 | 1 | 1 | 1 | 0 |
| Kalantri | 2005 | 1 | P | 1 | 1 | P | 1 | 1 | P | 1 | 1 | 1 | 1 | 1 | 1 | 1 | P | 1 | 1 | P | 1 | 1 | P | P | 1 | 1 | 1 | 1 |
| Karassa | 2006 | 1 | 1 | 1 | 1 | 0 | 1 | 1 | P | 0 | 0 | P | P | 1 | 1 | 1 | P | 0 | 1 | P | 0 | 1 | P | P | 1 | 1 | 1 | 0 |
| Karassa | 2005 | 1 | 1 | 1 | P | 0 | 1 | 1 | P | 0 | 1 | 1 | P | 1 | 1 | 1 | 1 | 1 | 1 | P | 1 | 1 | 1 | 1 | 1 | 1 | 1 | 1 |
| Kassai | 2004 | 1 | 1 | 1 | 1 | 0 | 1 | 1 | P | 1 | 1 | P | P | 1 | 1 | 1 | 0 | P | 1 | 1 | P | 1 | 1 | 0 | 1 | 0 | 1 | 0 |
| Kelly | 2001 | 1 | P | 1 | P | P | 1 | 1 | P | 0 | P | 1 | 1 | 1 | 1 | P | 1 | P | 0 | P | 0 | 1 | P | 1 | 1 | P | 1 | 1 |
| Khunti | 2004 | 1 | 1 | 1 | 1 | 0 | 1 | 1 | P | 0 | 1 | 0 | 0 | 1 | 1 | 0 | 0 | P | 1 | 0 | 1 | 1 | 0 | 0 | 1 | 1 | 1 | 0 |
| Koliopoulos | 2007 | 1 | P | 1 | 1 | 0 | 1 | 1 | 1 | 0 | 0 | 1 | 0 | 1 | 1 | 1 | 1 | 0 | 1 | 0 | P | 1 | 1 | P | 1 | 1 | 1 | 1 |
| Kraag | 1995 | 1 | P | 1 | 0 | 0 | 1 | 1 | P | 0 | 0 | 0 | P | 1 | 1 | P | 0 | P | 1 | P | 1 | 1 | 0 | 0 | 1 | 0 | 1 | 0 |
| Krug | 2008 | 1 | P | 1 | P | 0 | 1 | 1 | P | 1 | 0 | 1 | P | 1 | 1 | 1 | 1 | 1 | 1 | P | 1 | 1 | 1 | P | 1 | P | 1 | 0 |
| Kwee | 2008 | 1 | 1 | 1 | 1 | 0 | 1 | 1 | 1 | 1 | 1 | 1 | 1 | 1 | 1 | 1 | 1 | P | 1 | 1 | 1 | 1 | 1 | 1 | 1 | P | 1 | 0 |
|  |  | **1** | **2** | **3** | **4** | **5** | **6** | **7** | **8** | **9** | **10** | **11** | **12** | **13** | **14** | **15** | **16** | **17** | **18** | **19** | **20** | **21** | **22** | **23** | **24** | **25** | **26** | **27** |
| Kwee | 2007 | 1 | P | 1 | 1 | 0 | 1 | 1 | 1 | 1 | 0 | P | P | 1 | 1 | 1 | 0 | P | 1 | 1 | 1 | 1 | 1 | 1 | 1 | 1 | 1 | 1 |
| Lameris | 2008 | 1 | P | 1 | 1 | P | 1 | 1 | 1 | 1 | 1 | P | 1 | 1 | 1 | 1 | 0 | 1 | P | P | 1 | 1 | 1 | 0 | 1 | 1 | 1 | 0 |
| Leal | 2008 | 1 | P | 1 | 1 | 0 | 1 | 1 | P | P | 1 | P | P | 1 | 1 | 1 | P | 1 | P | P | 1 | 1 | 1 | 1 | 1 | 1 | 1 | 0 |
| Leeflang | 2008 | 1 | 1 | 1 | 1 | 0 | 1 | 1 | 1 | 1 | 1 | 1 | 1 | 1 | 1 | 0 | 1 | 1 | 1 | 1 | 1 | 1 | 0 | 1 | 1 | 1 | 1 | 1 |
| Liang | 2008^a^ | 1 | P | 1 | P | 0 | 1 | 1 | P | 1 | 1 | 1 | P | 1 | 1 | 1 | 1 | P | P | P | 1 | 1 | 1 | 1 | 1 | 1 | 1 | 1 |
| Liang | 2008^b^ | 1 | P | 1 | P | 0 | 1 | 1 | P | 1 | 1 | 1 | 1 | 1 | 1 | 1 | 1 | P | P | P | 1 | 1 | 1 | P | 1 | 0 | 1 | 1 |
| Ling | 2008^a^ | 1 | P | 1 | P | 0 | 1 | 1 | P | 1 | 1 | P | 1 | 1 | 1 | 1 | 1 | 1 | P | 1 | 1 | 1 | 1 | 1 | 1 | 1 | 1 | 0 |
| Ling | 2008^b^ | 1 | P | 1 | P | 0 | 1 | 1 | P | P | 1 | 0 | P | 1 | 1 | 1 | 0 | 1 | 1 | P | 1 | 1 | 1 | 0 | 1 | 1 | 1 | 0 |
| Liu | 2006 | 1 | P | 1 | 1 | 0 | 1 | 1 | 1 | 1 | 1 | 1 | 1 | 1 | 1 | 1 | 1 | 1 | 1 | 1 | 1 | 1 | 1 | 1 | 1 | 1 | 1 | 1 |
| Lysakowski | 2001 | 1 | 1 | 1 | P | P | 1 | 1 | P | 1 | P | P | 1 | 1 | 1 | 0 | 0 | 1 | 1 | 1 | P | 1 | 0 | 0 | 1 | 1 | 1 | 1 |
| Makrydimas | 2003 | 1 | 1 | 1 | 1 | 0 | 1 | 1 | P | 0 | 1 | 1 | 0 | 1 | 1 | 1 | 0 | 1 | 1 | 0 | 1 | 1 | P | 0 | 1 | 0 | 1 | 0 |
| Mant | 2004 | 1 | P | 1 | 1 | P | 1 | 1 | 1 | 1 | 1 | P | 0 | 1 | 1 | 1 | 0 | 1 | 1 | 0 | 0 | 1 | 1 | 0 | 1 | 1 | 1 | 1 |
| Martin | 2008 | 1 | P | 1 | P | 0 | 1 | 1 | P | 1 | 1 | 1 | 1 | 1 | 1 | 1 | 0 | 0 | P | 1 | 1 | 1 | 1 | 0 | 1 | 0 | 1 | 1 |
|  |  | **1** | **2** | **3** | **4** | **5** | **6** | **7** | **8** | **9** | **10** | **11** | **12** | **13** | **14** | **15** | **16** | **17** | **18** | **19** | **20** | **21** | **22** | **23** | **24** | **25** | **26** | **27** |
| Martin | 2007 | 1 | P | 1 | 1 | P | 1 | 1 | P | 1 | 1 | 1 | 1 | 1 | 1 | 1 | 0 | 0 | P | 1 | 1 | 1 | P | 0 | 1 | 0 | 1 | 1 |
| Martin | 2006 | 1 | P | 1 | 1 | P | 1 | 1 | 1 | 1 | 1 | 1 | 0 | 1 | 1 | P | 0 | 1 | 1 | 0 | 1 | 1 | 1 | 0 | 1 | P | 1 | 1 |
| Marx | 2005 | 1 | 1 | 1 | 1 | 0 | 1 | 1 | P | 0 | 1 | 1 | 1 | 1 | 1 | 1 | 0 | 1 | 1 | 1 | 0 | 1 | 1 | 0 | 1 | 1 | 1 | 0 |
| Medeiros | 2005 | 1 | 0 | P | P | 0 | 1 | 1 | P | P | 1 | 1 | 1 | 1 | 1 | 1 | 0 | 1 | P | 1 | 1 | 1 | 1 | 0 | 1 | 1 | 1 | 0 |
| Meijer | 2008 | 1 | P | 1 | 1 | P | 1 | 1 | P | 0 | 1 | 1 | 0 | 1 | 1 | 1 | 1 | 1 | 1 | 0 | 1 | 1 | 1 | 1 | 1 | 1 | 1 | 0 |
| Meserve | 2008 | 1 | P | 1 | P | 0 | P | 1 | P | 1 | P | P | 1 | 1 | 1 | 0 | P | 1 | P | P | 1 | 1 | 0 | P | 1 | 1 | 1 | 1 |
| Micames | 2007 | 1 | 1 | 1 | 1 | 0 | 1 | 1 | P | 1 | 1 | P | P | 1 | 1 | 0 | 1 | 1 | P | 0 | P | 1 | 1 | 1 | 1 | 0 | 1 | 1 |
| Mijnhout | 2001 | 1 | P | 1 | 1 | 0 | 1 | 1 | P | 1 | 1 | 1 | 1 | 1 | 1 | 1 | 1 | P | 1 | 1 | 1 | 1 | 1 | 1 | 1 | 1 | 1 | 0 |
| Mitchell | 2008 | 1 | P | 1 | P | 0 | P | 1 | P | P | 0 | 0 | 0 | 1 | 1 | 0 | 0 | 1 | 1 | 0 | 1 | 1 | 0 | 0 | 1 | 0 | 1 | 1 |
| Mol | 1998^a^ | 1 | P | 1 | P | 0 | 1 | 1 | P | 0 | P | 1 | 1 | 1 | 1 | 1 | 1 | P | P | 1 | P | 1 | 1 | 1 | 1 | 1 | 1 | 0 |
| Mol | 1998^b^ | 1 | P | 1 | 1 | 0 | P | 1 | P | 0 | P | 1 | 1 | 1 | 1 | 1 | 1 | P | P | 1 | P | 1 | 1 | 1 | 1 | 1 | 1 | 0 |
| Moles | 2002 | 1 | P | 1 | P | 0 | 1 | 1 | P | 0 | 0 | 0 | 0 | 1 | 1 | 0 | 0 | P | P | 0 | P | 1 | 0 | 0 | 1 | P | 1 | 0 |
| Morgan | 2005 | 1 | P | 1 | 1 | 0 | 1 | 1 | P | 1 | 1 | 1 | 1 | 1 | 1 | 0 | 0 | 1 | P | P | 1 | 1 | P | 0 | 1 | 1 | 1 | 1 |
|  |  | **1** | **2** | **3** | **4** | **5** | **6** | **7** | **8** | **9** | **10** | **11** | **12** | **13** | **14** | **15** | **16** | **17** | **18** | **19** | **20** | **21** | **22** | **23** | **24** | **25** | **26** | **27** |
| Morisson | 2008 | 1 | P | 1 | 1 | 0 | P | 1 | P | 0 | 0 | 1 | P | 1 | 1 | 1 | 0 | P | 0 | P | 1 | 1 | 1 | 0 | 1 | 0 | 1 | 0 |
| Mowatt | 2008 | 1 | P | 1 | 1 | 1 | 1 | 1 | 1 | 1 | 1 | P | 1 | 1 | 1 | 1 | 0 | 1 | 1 | 1 | 1 | 1 | 1 | 0 | 1 | 1 | 1 | 1 |
| Mowatt | 2004 | 1 | P | 1 | 1 | P | 1 | 1 | 1 | 1 | 1 | 1 | 1 | 1 | 1 | 1 | 0 | P | 1 | 1 | 1 | 1 | 1 | 0 | 1 | 1 | 1 | 1 |
| Muchow | 2008 | 1 | 1 | 1 | 1 | 0 | 1 | 1 | P | 1 | 0 | 0 | 0 | 1 | 1 | 1 | 0 | 1 | 1 | 0 | 1 | 1 | 1 | 0 | 1 | 1 | 1 | 0 |
| Mulhall | 2005 | 1 | 1 | 1 | 1 | 0 | 1 | 1 | P | 1 | 1 | 1 | 1 | 1 | 1 | 1 | 1 | 1 | 1 | 1 | 1 | 1 | 1 | 1 | 1 | 1 | 1 | 1 |
| Nallamothu | 2001 | 1 | 1 | 1 | 1 | 0 | 1 | 1 | P | 0 | 1 | 1 | P | 1 | 1 | 0 | 1 | P | 1 | 0 | P | 1 | 0 | 0 | 1 | 1 | 1 | 1 |
| Nandalur | 2007 | 1 | 1 | 1 | P | 0 | 1 | 1 | P | 0 | 1 | 0 | P | 1 | 1 | 1 | 0 | 1 | 1 | 1 | 1 | 1 | 1 | 0 | 1 | 1 | 1 | 0 |
| Nandalur | 2008 | 1 | 1 | 1 | P | 0 | 1 | 1 | P | 0 | 1 | 1 | P | 1 | 1 | 1 | 0 | P | 1 | P | 1 | 1 | 1 | 0 | 1 | 1 | 1 | 0 |
| Nayak | 2006 | 1 | 1 | 1 | 1 | 0 | 1 | 1 | 1 | 1 | 1 | 1 | P | 1 | 1 | 0 | 0 | 1 | 1 | 1 | 1 | 1 | 0 | 0 | 1 | 1 | 1 | 1 |
| Niemann | 2008 | 1 | P | 1 | P | P | 1 | 1 | 1 | 1 | 0 | 0 | P | 1 | 1 | 1 | 0 | 1 | P | 0 | 1 | 1 | 1 | 0 | 1 | 0 | 1 | 0 |
| Noguchi | 2005 | 1 | P | 1 | P | 0 | 1 | 1 | P | P | 1 | 0 | 0 | 1 | 1 | 1 | 1 | 0 | P | 0 | 1 | 1 | 1 | 1 | 1 | 1 | 1 | 0 |
| Numans | 2004 | 1 | 1 | 1 | P | 0 | 1 | 1 | P | 1 | 1 | 1 | P | 1 | 1 | 0 | 0 | 1 | 1 | P | 1 | 1 | 0 | 0 | 1 | 1 | 1 | 1 |
| Ogilvie | 2005 | 1 | P | 1 | 1 | 0 | 1 | 1 | P | P | 0 | 1 | 0 | 1 | 1 | 1 | 0 | P | 1 | 0 | 1 | 1 | 1 | 0 | 1 | 1 | 1 | 0 |
|  |  | **1** | **2** | **3** | **4** | **5** | **6** | **7** | **8** | **9** | **10** | **11** | **12** | **13** | **14** | **15** | **16** | **17** | **18** | **19** | **20** | **21** | **22** | **23** | **24** | **25** | **26** | **27** |
| Ola | 2003 | 1 | 1 | 1 | P | P | 1 | 1 | P | 0 | 1 | 1 | 1 | 1 | 1 | 1 | 0 | 1 | P | P | 1 | 1 | 1 | 0 | 1 | 0 | 1 | 1 |
| Owens | 1996 | 1 | P | 1 | 1 | 0 | 1 | 1 | 1 | 1 | 1 | 1 | P | 1 | 1 | P | 0 | P | 0 | P | 0 | 1 | P | 1 | 1 | 0 | 1 | 1 |
| Pai | 2004 | 1 | P | 1 | 1 | 0 | 1 | 1 | P | 1 | 1 | P | 1 | 1 | 1 | 1 | 1 | 1 | P | 1 | 1 | 1 | 1 | P | 1 | 1 | 1 | 1 |
| Pai | 2003 | 1 | P | 1 | P | 0 | 1 | 1 | P | 1 | 1 | P | 1 | 1 | 1 | 1 | 1 | 1 | 1 | 1 | 1 | 1 | 1 | 1 | 1 | 1 | 1 | 1 |
| Pai | 2005 | 1 | P | 1 | 1 | 0 | 1 | 1 | P | 1 | 1 | 1 | 1 | 1 | 1 | 1 | 1 | P | P | 0 | 1 | 1 | 1 | P | 1 | 1 | 1 | 1 |
| Pai | 2007 | 1 | P | P | 1 | 0 | 1 | 1 | P | 1 | 1 | 1 | 1 | 1 | 1 | 1 | 0 | 0 | P | 0 | 1 | 1 | 0 | 0 | 1 | 1 | 1 | 1 |
| Pakos | 2005 | 1 | 1 | 1 | 1 | 0 | 1 | 1 | P | 0 | 1 | 1 | P | 1 | 1 | 1 | 1 | 0 | 1 | P | 0 | 1 | 1 | 1 | 1 | 1 | 1 | 0 |
| Pakos | 2007^a^ | 1 | P | 1 | 1 | 0 | 1 | 1 | 1 | 1 | 1 | 1 | 1 | 1 | 1 | 1 | 0 | P | P | P | 1 | 1 | 1 | 0 | 1 | 1 | 1 | 0 |
| Pakos | 2007^b^ | 1 | P | 1 | 1 | 0 | 1 | 1 | P | 1 | 1 | 1 | P | 1 | 1 | 1 | 1 | 0 | 1 | P | 0 | 1 | 1 | 1 | 1 | 1 | 1 | 0 |
| Patwardhan | 2004 | 1 | 1 | 1 | 1 | P | 1 | 1 | P | 1 | 1 | P | P | 1 | 1 | 0 | 0 | P | 1 | P | 1 | 1 | P | P | 1 | P | 1 | 0 |
| Peters | 2008 | 1 | P | 1 | 1 | 0 | 1 | 1 | P | 1 | 1 | 1 | 1 | 1 | 1 | 1 | P | 1 | 1 | 1 | 1 | 1 | 0 | P | 1 | 1 | 1 | 0 |
| Pfeiffer | 2006 | 1 | P | 1 | 1 | 0 | P | 1 | P | 0 | 0 | 1 | P | 1 | 1 | 1 | 1 | 1 | 1 | P | 1 | 1 | 1 | 1 | P | 1 | 1 | 1 |
| Pirozzo | 2003 | 1 | 1 | 1 | 1 | 0 | 1 | 1 | 1 | 1 | 1 | 1 | 1 | 1 | 1 | 0 | 0 | 1 | 1 | 1 | 1 | 1 | 0 | 0 | 1 | P | 1 | 1 |
|  |  | **1** | **2** | **3** | **4** | **5** | **6** | **7** | **8** | **9** | **10** | **11** | **12** | **13** | **14** | **15** | **16** | **17** | **18** | **19** | **20** | **21** | **22** | **23** | **24** | **25** | **26** | **27** |
| Price | 2005 | 1 | P | 1 | 1 | 0 | 1 | 1 | P | P | 0 | 0 | 0 | 1 | 1 | 1 | 0 | 1 | 1 | 0 | 1 | 1 | 1 | 0 | 1 | 1 | 1 | 0 |
| Puli | 2008^a^ | 1 | 1 | 1 | 1 | 0 | 1 | 1 | P | P | 1 | 0 | P | 1 | 1 | 1 | 0 | 1 | P | 0 | 1 | 1 | 1 | 1 | 1 | 0 | 1 | 0 |
| Puli | 2008^b^ | 1 | 1 | 1 | P | 0 | 1 | 1 | P | P | 1 | 0 | 0 | 1 | 1 | 1 | 0 | 1 | P | 0 | 1 | 1 | 1 | 0 | 1 | P | 1 | 0 |
| Puli | 2008^c^ | 1 | P | 1 | P | 0 | 1 | 1 | P | P | 1 | 0 | 0 | 1 | 1 | 1 | 0 | 1 | P | 0 | 1 | 1 | 1 | 0 | 1 | 0 | 1 | 0 |
| Puli | 2008^d^ | 1 | P | 1 | P | 0 | 1 | 1 | P | P | 1 | P | 0 | 1 | 1 | 1 | 0 | 1 | P | 0 | 1 | 1 | 1 | 0 | 1 | P | 1 | 0 |
| Puli | 2007 | 1 | P | 1 | 1 | 0 | 1 | 1 | P | 1 | 1 | 0 | 0 | 1 | 1 | 1 | 0 | P | P | 0 | 1 | 1 | 1 | 0 | 1 | 0 | 1 | 1 |
| Purkayastha | 2007^a^ | 1 | P | 1 | 1 | 0 | 1 | 1 | P | 0 | 1 | 1 | 1 | 1 | 1 | 1 | 1 | 0 | 1 | 1 | 1 | 1 | 1 | 1 | 1 | 1 | 1 | 1 |
| Purkayastha | 2006 | 1 | P | 1 | 1 | 0 | 1 | 1 | P | 0 | 1 | 1 | 1 | 1 | 1 | 1 | 1 | 0 | 1 | 1 | 1 | 1 | 1 | 1 | 1 | 1 | 1 | 0 |
| Purkayastha | 2005 | 1 | P | 1 | 1 | 0 | 1 | 1 | P | 0 | 1 | 1 | P | 1 | 1 | 1 | 1 | 0 | 1 | P | 1 | 1 | 1 | 1 | 1 | P | 1 | 1 |
| Purkayastha | 2007^b^ | 1 | P | 1 | P | 0 | P | 1 | P | 0 | 1 | 1 | 1 | 1 | 1 | 1 | 1 | 0 | 1 | 1 | 1 | 1 | 1 | 1 | 1 | 0 | 1 | 1 |
| Reese | 2006 | P | P | 1 | 1 | 0 | 1 | 1 | P | 1 | 1 | P | P | 1 | 1 | 1 | 1 | P | P | P | 1 | 1 | 1 | P | 1 | 0 | 1 | 0 |
| Roddam | 2005 | 1 | P | 1 | 1 | 0 | 1 | 1 | P | 0 | 1 | 1 | 1 | 1 | 1 | 1 | 1 | 0 | 1 | 1 | 0 | 1 | 1 | 1 | 1 | 1 | 1 | 0 |
| Rodgers | 2006 | 1 | P | 1 | 1 | P | 1 | 1 | 1 | 1 | 1 | 1 | 1 | 1 | 1 | 1 | 1 | 1 | P | 1 | 1 | 1 | 1 | 1 | 1 | P | 1 | 1 |
|  |  | **1** | **2** | **3** | **4** | **5** | **6** | **7** | **8** | **9** | **10** | **11** | **12** | **13** | **14** | **15** | **16** | **17** | **18** | **19** | **20** | **21** | **22** | **23** | **24** | **25** | **26** | **27** |
| Ross | 2000 | 1 | P | 1 | P | P | 1 | 1 | P | 0 | 1 | 1 | P | 1 | 1 | 0 | 0 | P | P | 0 | 0 | 1 | 0 | 0 | 1 | 1 | 1 | 1 |
| Roy | 2005 | 1 | 1 | 1 | P | 0 | 1 | 1 | 1 | P | 1 | 1 | 0 | 1 | 1 | 1 | 0 | 1 | P | 0 | 1 | 1 | 1 | 0 | 1 | 1 | 1 | 1 |
| Safdar | 2005 | 1 | P | 1 | P | 0 | 1 | 1 | P | 0 | 0 | 1 | 1 | 1 | 1 | 1 | 1 | 1 | 1 | 1 | 1 | 1 | 1 | 1 | 1 | 1 | 1 | 1 |
| Samson | 2002 | 1 | P | 1 | 1 | P | 1 | 1 | P | 0 | 0 | P | 1 | 1 | 1 | 0 | 0 | P | 1 | P | 1 | 1 | 0 | 0 | 1 | 0 | 1 | 1 |
| Sarmiento | 2003 | 1 | P | 1 | 1 | 0 | 1 | 1 | P | 1 | 1 | 1 | 1 | 1 | 1 | 1 | 1 | P | P | 1 | 0 | 1 | 1 | 1 | 1 | P | 1 | 1 |
| Sauerland | 2004 | 1 | P | 1 | P | 0 | 1 | 1 | P | 0 | 0 | P | P | 1 | 1 | 0 | 0 | P | 1 | P | 1 | 1 | 0 | 0 | 1 | 1 | 1 | 0 |
| Scholten | 2001 | 1 | 1 | 1 | 1 | 0 | 1 | 1 | P | 1 | 1 | P | P | 1 | 1 | 1 | 1 | P | 1 | 0 | P | 1 | 1 | P | 1 | 1 | 1 | 0 |
| Scholten | 2003 | 1 | 1 | 1 | P | 0 | 1 | 1 | P | P | P | P | 1 | 1 | 1 | 1 | 1 | P | 1 | 1 | 1 | 1 | 1 | 1 | 1 | 0 | P | 0 |
| Schreiber | 2003 | P | P | 1 | 1 | 0 | 1 | 1 | P | 0 | 0 | 0 | 0 | 0 | P | 0 | 0 | P | P | 0 | P | P | 0 | 0 | 1 | 1 | 1 | 0 |
| Selman | 2005 | 1 | P | 1 | 1 | 0 | P | 1 | P | 1 | P | P | 1 | 1 | 1 | 0 | 0 | 1 | P | 1 | 1 | 1 | 1 | 0 | 1 | 0 | 1 | 0 |
| Selman | 2008^a^ | 1 | P | 1 | 1 | 0 | 1 | 1 | 1 | 1 | 0 | 1 | 1 | 1 | 1 | 1 | 1 | 1 | 1 | 1 | 1 | 1 | 1 | 1 | 1 | 1 | 1 | 1 |
| Selman | 2008^b^ | 1 | P | 1 | P | 0 | 1 | 1 | 1 | 1 | 0 | 1 | 1 | 1 | 1 | 1 | 0 | 1 | 1 | 1 | 0 | 1 | 1 | 0 | 1 | 0 | 1 | 1 |
| Shafiq | 2005 | 1 | P | 1 | 1 | 0 | P | 1 | P | 1 | 0 | 0 | 0 | 1 | 1 | 1 | 0 | P | 0 | 0 | 1 | 1 | 1 | 0 | 1 | P | 1 | 0 |
|  |  | **1** | **2** | **3** | **4** | **5** | **6** | **7** | **8** | **9** | **10** | **11** | **12** | **13** | **14** | **15** | **16** | **17** | **18** | **19** | **20** | **21** | **22** | **23** | **24** | **25** | **26** | **27** |
| Shaheen | 2008 | 1 | P | 1 | 1 | 0 | 1 | 1 | P | 1 | 1 | P | 1 | 1 | 1 | 1 | 1 | P | 1 | 1 | 1 | 1 | 1 | 1 | 1 | 1 | 1 | 1 |
| Shaheen | 2007^a^ | 1 | P | 1 | 1 | 0 | 1 | 1 | P | 1 | 1 | 1 | 1 | 1 | 1 | 1 | 1 | P | 1 | 1 | 0 | 1 | 1 | 1 | 1 | 1 | 1 | 0 |
| Shaheen | 2007^b^ | 1 | P | 1 | 1 | 0 | 1 | 1 | P | 1 | 1 | P | 1 | 1 | 1 | 1 | 1 | P | 1 | P | 0 | 1 | P | 1 | 1 | 1 | 1 | 1 |
| Shi | 2008 | 1 | P | 1 | P | 0 | 1 | 1 | P | 1 | 1 | 1 | P | 1 | 1 | 1 | 1 | P | 0 | P | 1 | 1 | 1 | P | 1 | 1 | 1 | 0 |
| Shie | 2008 | 1 | P | 1 | 1 | 0 | 1 | 1 | 1 | 0 | 1 | 1 | 0 | 1 | 1 | 0 | 0 | 1 | 1 | 0 | P | 1 | 0 | 0 | 1 | 1 | 1 | 0 |
| Shiga | 2006 | 1 | P | 1 | P | 0 | 1 | 1 | P | P | 0 | 1 | P | 1 | 1 | 1 | 0 | 1 | 1 | P | 1 | 1 | P | 0 | 1 | 1 | 1 | 1 |
| Song | 2005 | 1 | 1 | 1 | 1 | 0 | 1 | 1 | P | 0 | 1 | 0 | 0 | 1 | 1 | 1 | 0 | 0 | P | 0 | 1 | 1 | 1 | 0 | 1 | P | 1 | 0 |
| Sosna | 2008 | 0 | P | 1 | P | 0 | 1 | 1 | P | 0 | 1 | 1 | 0 | 1 | 1 | 1 | 0 | P | 1 | 0 | P | 1 | 1 | 0 | 1 | 1 | 1 | 0 |
| Sotiriadis | 2003 | 1 | 1 | 1 | 1 | 0 | 1 | 1 | P | 0 | 1 | P | 0 | 1 | 1 | 1 | 0 | P | 1 | 0 | 1 | 1 | 1 | 0 | 1 | 0 | 1 | 0 |
| Speight | 2006 | P | 1 | 1 | P | P | 1 | 1 | 1 | 1 | 1 | 1 | 0 | 1 | 1 | 0 | 1 | P | 1 | 0 | 1 | 1 | 0 | 0 | 1 | 1 | 1 | 1 |
| St John | 2006 | 1 | P | 1 | 0 | 0 | 1 | 1 | P | 1 | 0 | P | 0 | 1 | 1 | 1 | 0 | P | 1 | P | P | 1 | P | 1 | 1 | P | 1 | 0 |
| Stein | 2006 | 1 | P | 1 | P | 0 | 1 | 1 | P | 0 | 0 | 1 | 0 | 1 | 1 | 0 | 0 | 1 | 1 | 0 | 1 | 1 | 0 | 0 | 1 | 0 | 1 | 0 |
| Stein | 2004 | 1 | 1 | 1 | 1 | 0 | 1 | 1 | P | 1 | 1 | P | P | 1 | 1 | P | 1 | 1 | 1 | P | 1 | 1 | 0 | 1 | 1 | 0 | 1 | 0 |
|  |  | **1** | **2** | **3** | **4** | **5** | **6** | **7** | **8** | **9** | **10** | **11** | **12** | **13** | **14** | **15** | **16** | **17** | **18** | **19** | **20** | **21** | **22** | **23** | **24** | **25** | **26** | **27** |
| Steingart | 2007^a^ | 1 | P | 1 | 1 | 0 | 1 | 1 | P | 1 | 1 | 1 | P | 1 | 1 | P | P | 1 | P | P | 1 | 1 | P | P | 1 | 1 | 1 | 1 |
| Steingart | 2007^b^ | 1 | P | 1 | 1 | 0 | 1 | 1 | P | 1 | 1 | 1 | P | 1 | 1 | 0 | 1 | 1 | P | 0 | 1 | 1 | 0 | 1 | 1 | 0 | 1 | 1 |
| Steingart | 2006 | 1 | 0 | 1 | 1 | 0 | 1 | 1 | P | 1 | 1 | 1 | 1 | 1 | 1 | 0 | P | 1 | P | 0 | 1 | 1 | 0 | P | 1 | 1 | 1 | 1 |
| Stengel | 2005 | 1 | P | 1 | 1 | 0 | 1 | 1 | 1 | 1 | 1 | 1 | 1 | 1 | 1 | 1 | 1 | 1 | 1 | 0 | 1 | 1 | 1 | 1 | 1 | 1 | 1 | 1 |
| Stengel | 2001 | 1 | P | 1 | P | 0 | P | 1 | P | 0 | 0 | P | P | 1 | 1 | 1 | 1 | P | P | P | 1 | 1 | 1 | 1 | 1 | 0 | 1 | 0 |
| Takata | 2003 | P | P | 1 | 1 | P | P | 1 | P | 1 | 0 | 0 | 1 | 1 | 1 | 0 | 0 | 1 | 1 | 1 | 0 | 1 | 0 | 0 | 1 | 1 | 1 | 0 |
| Tang | 2007 | 1 | P | 1 | 1 | 0 | 1 | 1 | P | 0 | 1 | 1 | 1 | 1 | 1 | 1 | 1 | 1 | 1 | 1 | 1 | 1 | 1 | 1 | 1 | P | 1 | 1 |
| Terasawa | 2004 | 1 | 1 | 1 | 1 | 0 | 1 | 1 | P | 1 | 1 | 1 | 1 | 1 | 1 | 1 | 1 | 1 | 1 | P | 1 | 1 | 1 | P | 1 | 1 | 1 | 1 |
| Termaat | 2005 | 1 | P | 1 | P | 0 | 1 | 1 | 1 | 1 | 1 | 0 | 1 | 1 | 1 | 1 | 1 | 0 | 1 | 1 | 1 | 1 | 1 | 1 | 1 | 0 | 1 | 1 |
| Tew | 2005 | 1 | P | 1 | P | P | 1 | 1 | 1 | 1 | 1 | 0 | 1 | 1 | 1 | 1 | 1 | P | 1 | 1 | 1 | 1 | 1 | 1 | 1 | 0 | 1 | 0 |
| Trochez-Martinez | 2007 | 1 | 1 | 1 | 1 | 0 | 1 | 1 | P | 0 | 0 | P | P | 1 | 1 | 1 | 1 | 1 | 1 | P | P | 1 | 1 | 1 | P | 0 | 1 | 0 |
| Trowbridge | 2003 | P | 1 | 1 | P | 0 | 1 | 1 | P | 1 | 1 | P | 0 | 1 | 1 | 0 | 0 | P | 1 | P | 0 | 1 | 0 | 0 | 1 | 1 | 1 | 1 |
| Tse | 2008 | 1 | 1 | 1 | P | 0 | 1 | 1 | P | 1 | 1 | 1 | 1 | 1 | 1 | 1 | 1 | 1 | 1 | P | 1 | 1 | 1 | 1 | 1 | 1 | 1 | 1 |
|  |  | **1** | **2** | **3** | **4** | **5** | **6** | **7** | **8** | **9** | **10** | **11** | **12** | **13** | **14** | **15** | **16** | **17** | **18** | **19** | **20** | **21** | **22** | **23** | **24** | **25** | **26** | **27** |
| Tuon | 2006 | 1 | 1 | 1 | 1 | 0 | 1 | 1 | P | 1 | 0 | P | P | 1 | 1 | 1 | 0 | 1 | 0 | P | 1 | 1 | 1 | 0 | 1 | 0 | 1 | 0 |
| Tuon | 2007 | 1 | 1 | 1 | 1 | 0 | 1 | 1 | P | 1 | 1 | 1 | 1 | 1 | 1 | 1 | 0 | 1 | P | 0 | 1 | 1 | 1 | 0 | 1 | 0 | P | 0 |
| Vakil | 2006 | 1 | 1 | 1 | 1 | 0 | 1 | 1 | P | 1 | 1 | P | 0 | 1 | 1 | 0 | 0 | 1 | P | P | 1 | 1 | 1 | 0 | 1 | 0 | 1 | 0 |
| van Dongen | 2007 | 1 | 1 | 1 | 1 | 0 | 1 | 1 | 1 | 1 | 0 | P | P | 1 | 1 | 1 | 0 | 1 | P | P | 1 | 1 | 1 | 0 | 1 | P | 1 | 0 |
| van Randen | 2008 | 1 | 1 | 1 | 1 | 0 | 1 | 1 | 1 | 1 | 1 | 1 | 1 | 1 | 1 | 0 | 0 | P | 1 | P | 1 | 1 | 0 | 0 | 1 | 1 | 1 | 0 |
| van Westreenen | 2004 | 1 | P | 1 | 1 | 0 | 1 | 1 | P | 1 | 0 | P | 1 | 1 | 1 | 0 | 0 | P | 1 | 1 | 1 | 1 | 0 | 0 | 1 | 1 | 1 | 1 |
| van Zaane | 2008 | 1 | P | 1 | P | 0 | 1 | 1 | 1 | 0 | 1 | 1 | 1 | 1 | 1 | 1 | 1 | P | 1 | P | 1 | 1 | 1 | P | 1 | 1 | 1 | 1 |
| Vanezis | 2008 | P | P | 1 | 1 | 0 | 1 | 1 | P | 0 | 0 | 0 | 0 | 1 | 1 | 0 | 0 | 0 | 1 | P | 0 | 1 | 0 | 0 | 1 | 1 | 1 | 0 |
| Vanhoenacker | 2007 | 1 | 1 | 1 | 1 | 0 | 1 | 1 | 1 | P | 1 | P | P | 1 | 1 | 1 | 1 | 1 | 1 | 1 | 1 | 1 | 1 | 1 | 1 | 1 | 1 | 1 |
| Vasbinder | 2001 | 1 | P | 1 | 1 | P | 1 | 1 | P | 1 | 0 | 1 | P | 1 | 1 | 0 | 0 | P | 0 | P | 1 | 1 | 0 | 0 | 1 | P | 1 | 1 |
| Vestergaard | 2008 | 1 | P | 1 | 1 | 0 | 1 | 1 | P | 1 | 0 | 1 | 0 | 1 | 1 | 0 | 0 | P | 1 | 0 | 1 | 1 | 0 | 0 | 1 | 0 | 1 | 1 |
| Virgili | 2007 | 1 | 1 | 1 | 1 | 0 | 1 | 1 | 1 | 1 | 0 | 0 | 1 | 1 | 1 | 1 | 0 | 0 | 1 | 1 | 1 | 1 | 1 | 0 | 1 | 1 | 1 | 0 |
| Vlaar | 2007 | 1 | 1 | 1 | 1 | 0 | 1 | 1 | P | 1 | 0 | P | P | 1 | 1 | 1 | 0 | P | P | 0 | 1 | 1 | 1 | 0 | 1 | P | 1 | 1 |
|  |  | **1** | **2** | **3** | **4** | **5** | **6** | **7** | **8** | **9** | **10** | **11** | **12** | **13** | **14** | **15** | **16** | **17** | **18** | **19** | **20** | **21** | **22** | **23** | **24** | **25** | **26** | **27** |
| von Roon | 2007 | P | P | 1 | 1 | 0 | 1 | 1 | P | 0 | 1 | 1 | 0 | 1 | 1 | 1 | 1 | 1 | 1 | 0 | P | 1 | 1 | 1 | 1 | P | 1 | 0 |
| Vroomen | 1999 | 1 | P | 1 | P | 0 | P | 1 | P | 0 | P | 1 | 1 | 1 | 1 | 0 | 0 | 0 | P | 1 | 0 | 1 | 0 | 0 | 1 | 1 | 1 | 0 |
| Wang | 2008 | 1 | 1 | 1 | P | 0 | 1 | 1 | 1 | 0 | 0 | 1 | P | 1 | 1 | 1 | 0 | P | P | P | 1 | 1 | 1 | 0 | 1 | 1 | 1 | 0 |
| Wang | 2005 | 1 | P | 1 | 1 | 0 | 1 | 1 | P | 1 | 1 | 0 | P | 1 | 1 | 1 | 1 | 1 | P | 0 | 1 | 1 | 1 | 1 | 1 | 1 | 1 | 1 |
| Wang | 2006 | 1 | P | 1 | 1 | 0 | 1 | 1 | P | 1 | P | P | P | 1 | 1 | 1 | 0 | 1 | P | P | 1 | 1 | 1 | 0 | 1 | 0 | 1 | 0 |
| Wardlaw | 2006 | 1 | P | 1 | 1 | P | 1 | 1 | 1 | P | 1 | 1 | 1 | 1 | 1 | 1 | 0 | 1 | P | P | 1 | 1 | 1 | 0 | 1 | 1 | 1 | 0 |
| White | 2000 | 1 | P | 1 | P | 0 | 1 | 1 | P | P | 1 | 1 | P | 1 | 1 | P | 1 | P | P | P | 0 | 1 | P | 1 | 1 | 0 | 1 | 0 |
| Whiting | 2006^a^ | 1 | P | 1 | 1 | 0 | 1 | P | P | 1 | 1 | 0 | 1 | 1 | 1 | 0 | 0 | 1 | 1 | 1 | P | 1 | 0 | 0 | 1 | 1 | 1 | 1 |
| Whiting | 2006^b^ | 1 | 1 | 1 | 1 | P | 1 | 1 | 1 | 1 | 1 | 1 | 1 | 1 | 1 | 1 | 1 | 1 | 1 | 1 | 1 | 1 | 1 | 1 | 1 | 0 | 1 | 1 |
| Whitsel | 2000 | 1 | P | 1 | 1 | 0 | 1 | 1 | P | P | 0 | 1 | 0 | 1 | 1 | P | 0 | P | 1 | P | P | 1 | P | 0 | 1 | 1 | 1 | 1 |
| Will | 2006 | 1 | P | 1 | P | 0 | P | 1 | P | P | 1 | 1 | 1 | 1 | 1 | 1 | 1 | 1 | 1 | 0 | 1 | 1 | 1 | 1 | 1 | 1 | 1 | 1 |
| Williams | 2007 | P | 1 | 1 | 1 | P | 1 | 1 | 1 | 1 | 0 | P | 1 | 1 | 1 | 1 | 1 | 1 | 1 | 1 | 0 | 1 | 1 | 1 | 1 | 0 | 1 | 1 |
| Wittkampf | 2007 | 1 | 1 | 1 | 1 | 0 | 1 | 1 | P | 1 | 0 | P | 1 | 1 | 1 | 0 | 0 | 1 | 1 | P | 1 | 1 | 1 | 0 | 1 | 1 | 1 | 0 |
|  |  | **1** | **2** | **3** | **4** | **5** | **6** | **7** | **8** | **9** | **10** | **11** | **12** | **13** | **14** | **15** | **16** | **17** | **18** | **19** | **20** | **21** | **22** | **23** | **24** | **25** | **26** | **27** |
| Worster | 2008 | 1 | P | 1 | 1 | P | P | 1 | P | 0 | 1 | P | 1 | 1 | 1 | 1 | 0 | 1 | 1 | P | 1 | 1 | 1 | 0 | 1 | 1 | 1 | 1 |
| Worster | 2002 | 1 | P | 1 | P | 0 | 1 | 1 | P | 1 | 0 | P | 0 | 1 | 1 | P | 0 | 1 | 0 | 1 | 1 | 1 | 1 | 0 | 1 | 0 | 1 | 1 |
| Wykes | 2004 | 1 | 0 | 1 | P | 0 | P | 1 | P | 1 | 0 | P | 1 | 1 | 1 | 1 | 0 | 1 | P | P | 1 | 1 | 1 | 0 | 1 | P | 1 | 0 |

NOTES

Individual items 1-27 as defined in PRISMA statement

1 = Compliant with PRISMA item

0 = Non-compliant with PRISMA item

P = Partially compliant with PRISMA item
