# Supplementary material for: The assessment of the quality of reporting of meta-analyses in diagnostic research: a systematic review
Source: BMC Med Res Methodol. 2011 Dec 9;11:163. doi: 10.1186/1471-2288-11-163 (PMC3258221; doi:10.1186/1471-2288-11-163)
Supplement: Additional file 4 — Appendix 4 - Evidence Tables. [file 1471-2288-11-163-S4.DOC]

**Appendix 4 Evidence Tables – see notes at end of table for column headings**

| **STUDY** |  | **A** | **B** | **C** | **D** | **E** | **F** | **G** |
| --- | --- | --- | --- | --- | --- | --- | --- | --- |
| Abdulla | 2007 | yes |  | yes |  |  |  | yes |
| Abubakar | 2007 | yes | yes | yes |  | yes |  | yes |
| Akcil | 2008 | yes |  | yes |  |  |  |  |
| Arbyesn | 2004 | yes |  | yes | yes |  |  |  |
| Arbyesn | 2008 | yes |  |  |  | yes |  | yes |
| Atieh | 2008 | yes |  | yes |  |  |  |  |
| Bafounta | 2004 | yes |  | yes |  |  |  | yes |
| Bafounta | 2001 | yes |  | yes |  |  |  |  |
| Bagai | 2006 | yes |  |  |  |  |  |  |
| Bakis | 2004 | yes |  |  | yes |  |  |  |
| Barnes | 2002 | yes |  | yes |  |  |  |  |
| Bastian | 1998 | yes |  |  |  |  |  |  |
| Benjaminse | 2006 | yes | yes | yes | yes |  |  |  |
| Berner | 2007 | yes |  | yes |  |  | yes |  |
| Berryes | 2002 | yes | yes | yes |  |  |  | yes |
| Berryes | 1999 | yes | yes | yes | yes |  |  | yes |
| Bipat | 2005 | yes |  | yes |  |  |  | yes |
| Bipat | 2004 | yes |  | yes | yes |  |  | yes |
| Bipat | 2003 | yes |  |  | yes |  |  | yes |
| Blacksell | 2006 | yes |  | yes |  |  | yes |  |
| Brealeyes | 2005 | yes |  | yes |  |  |  | yes |
| Brown | 2003 | yes |  | yes |  | yes | yes | yes |
| Brown | 2002 | yes |  |  |  | yes |  |  |
| Bruyesninckx | 2008 | yes | yes | yes |  | yes | yes | yes |
| Burr | 2007 | yes | yes | yes |  | yes |  | yes |
| Campens | 1997 | yes |  | yes |  |  |  |  |
| Castilla-Rilo | 2007 | yes |  | yes |  |  |  |  |
| Cavallazzi | 2008 | yes |  |  |  |  |  | yes |
| Cepoiu | 2008 | yes |  | yes | yes |  | yes |  |
| Chalco | 2005 | yes | yes | yes | yes | yes |  |  |
| Chen | 2001 | yes |  | yes |  |  |  | yes |
| Chua | 2008 | yes |  |  |  |  |  |  |
| Clark | 2001 | yes |  |  |  | yes |  | yes |
| Clark | 2002 | yes |  | yes |  | yes |  | yes |
| Clark | 2000 | yes |  |  |  |  |  |  |
| Colin | 2001 | yes |  |  |  |  |  |  |
| Dales | 1990 | yes |  |  |  |  |  |  |
| de Bondt | 2007 | yes |  | yes | yes |  |  | yes |
| de Kroon | 2003 | yes |  | yes |  | yes |  | yes |
| Debreyes | 2008 | yes |  | yes | yes |  |  |  |
| Delgado-Bolton | 2003 | yes | yes |  | yes | yes |  | yes |
| Deville | 2004 | yes | yes |  |  |  |  | yes |
| Deville | 2000 | yes |  |  |  |  |  |  |
| **STUDY** |  | **A** | **B** | **C** | **D** | **E** | **F** | **G** |
| Di Nisio | 2007 | yes |  |  |  | yes | yes | yes |
| Di Fabio | 1996 | yes | yes |  |  |  |  |  |
| Dinh | 2008 | yes |  |  | yes |  |  |  |
| Dinnes | 2007 | yes | yes | yes |  | yes |  | yes |
| Dinnes | 2003 | yes | yes | yes |  |  |  | yes |
| Dong | 2008 | yes |  | yes | yes | yes |  | yes |
| Doria | 2006 | yes |  | yes | yes | yes |  | yes |
| Dubin | 2005 | yes |  | yes |  |  | yes | yes |
| Ebell | 2004 |  |  | yes |  | yes |  |  |
| Engelbrecht | 2002 | yes |  |  |  |  |  |  |
| Ewald | 2004 | yes |  |  |  |  |  |  |
| Ewald | 2008 | yes | yes |  |  |  |  |  |
| Fancher | 2004 | yes | yes |  |  | yes |  |  |
| Fischer | 2001 | yes |  |  |  |  |  |  |
| Flores | 2005 | yes |  | yes | yes |  |  |  |
| Ford | 2008 | yes |  | yes |  |  |  |  |
| Fraquelli | 2005 | yes | yes | yes |  | yes |  | yes |
| Friedrich-Rust | 2008 | yes |  | yes |  |  |  |  |
| Geifman-Holtzman | 2006 | yes |  | yes |  |  |  |  |
| Gisbert | 2006 | yes |  | yes |  |  |  | yes |
| Glas | 2003 | yes |  |  |  |  |  |  |
| Goodacre | 2006 | yes | yes | yes |  | yes |  |  |
| Gordon | 2003 | yes |  |  |  |  | yes |  |
| Goto | 2003 | yes |  |  |  | yes |  | yes |
| Gould | 2003 | yes | yes | yes |  |  | yes | yes |
| Gu | 2007 | yes |  | yes | yes | yes |  | yes |
| Gupta | 2002 | yes |  |  |  | yes |  | yes |
| Hallan | 1997 |  |  |  |  |  |  |  |
| Halligan | 2005 | yes |  | yes |  | yes |  | yes |
| Hamon | 2008 | yes |  | yes | yes | yes |  | yes |
| Hamon | 2008 | yes |  | yes | yes |  |  | yes |
| Hancock | 2007 | yes | yes | yes | yes |  |  |  |
| Hayesashino | 2005 | yes |  |  | yes |  |  | yes |
| Hegedus | 2007 | yes | yes | yes |  | yes |  |  |
| Hegedus | 2008 | yes | yes | yes |  | yes |  | yes |
| Heijenbrok-Kal | 2007 ^b^ | yes |  | yes |  | yes |  | yes |
| Heijenbrok-Kal | 2007^a^ | yes |  |  | yes |  |  |  |
| Hobbyes | 2001 | yes | yes |  | yes |  |  |  |
| Hofman | 2000 | yes |  | yes |  |  |  |  |
| Holmes | 2007 | yes |  |  |  | yes |  |  |
| Holroyesd-Leduc | 2008 | yes |  |  |  | yes |  | yes |
| Holtyes | 2005 | yes | yes |  | yes |  | yes |  |
| Horsthuis | 2008 | yes | yes | yes | yes |  | yes | yes |
| Hovels | 2008 | yes |  |  |  | yes |  |  |
| Huicho | 2002 | yes | yes |  | yes | yes |  | yes |
| **STUDY** |  | **A** | **B** | **C** | **D** | **E** | **F** | **G** |
| Ioannidis | 2003 | yes |  |  |  |  |  |  |
| Jahromi | 2005 |  |  | yes | yes | yes |  | yes |
| Jiang | 2007 | yes | yes | yes |  | yes |  | yes |
| Jones | 2005 | yes |  | yes |  |  |  | yes |
| Joshi | 2007 | yes | yes |  | yes | yes |  | yes |
| Kalantri | 2005 | yes |  | yes |  | yes | yes | yes |
| Karassa | 2006 | yes |  | yes |  |  |  | yes |
| Karassa | 2005 | yes |  | yes |  |  |  | yes |
| Kassai | 2004 | yes |  | yes |  | yes |  |  |
| Kellyes | 2001 | yes |  | yes |  |  |  |  |
| Khunti | 2004 | yes |  | yes |  |  |  |  |
| Koliopoulos | 2007 | yes |  | yes | yes |  |  |  |
| Kraag | 1995 |  |  |  |  |  |  |  |
| Krug | 2008 | yes |  |  | yes | yes |  |  |
| Kwee | 2007 | yes | yes |  |  | yes |  | yes |
| Kwee | 2008 | yes | yes |  |  | yes |  | yes |
| Lameris | 2008 | yes | yes | yes | yes |  |  |  |
| Leal | 2008 | yes |  | yes | yes |  |  |  |
| Leeflang | 2008 | yes | yes | yes | yes | yes | yes | yes |
| Liang | 2008^b^ | yes |  | yes |  | yes |  | yes |
| Liang | 2008^a^ | yes |  | yes |  | yes |  | yes |
| Ling | 2008^b^ | yes |  | yes |  |  | yes |  |
| Ling | 2008^a^ | yes |  | yes |  |  |  |  |
| Liu | 2006 | yes |  | yes |  |  |  | yes |
| Lyessakowski | 2001 | yes |  | yes |  |  | yes |  |
| Makryesdimas | 2003 | yes |  |  |  |  |  |  |
| Mant | 2004 | yes | yes | yes |  |  |  | yes |
| Martin | 2007 | yes |  | yes |  | yes |  | yes |
| Martin | 2006 | yes | yes | yes |  |  |  | yes |
| Martin | 2008 | yes |  | yes |  | yes |  | yes |
| Marx | 2005 | yes |  | yes |  |  | yes | yes |
| Medeiros | 2005 | yes |  | yes |  |  |  | yes |
| Meijer | 2008 | yes |  |  |  |  |  | yes |
| Meserve | 2008 | yes | yes | yes |  |  |  | yes |
| Micames | 2007 | yes |  |  | yes | yes | yes | yes |
| Mijnhout | 2001 | yes |  | yes | yes | yes |  | yes |
| Mitchell | 2008 | yes |  | yes |  |  | yes |  |
| Mol | 1998^b^ | yes |  |  |  |  |  |  |
| Mol | 1998^a^ | yes |  |  |  |  |  | yes |
| Moles | 2002 | yes |  | yes | yes |  |  |  |
| Morgan | 2005 | yes |  | yes | yes | yes |  | yes |
| Morisson | 2008 | yes |  | yes |  |  |  |  |
| Mowatt | 2004 | yes | yes | yes |  |  |  | yes |
| Mowatt | 2008 | yes | yes | yes |  | yes |  | yes |
| Muchow | 2008 | yes |  | yes | yes |  |  |  |
| **STUDY** |  | **A** | **B** | **C** | **D** | **E** | **F** | **G** |
| Mulhall | 2005 | yes |  | yes |  | yes | yes | yes |
| Nallamothu | 2001 | yes |  |  | yes |  |  | yes |
| Nandalur | 2007 | yes |  |  | yes |  |  |  |
| Nandalur | 2008 | yes |  |  |  |  |  | yes |
| Nayesak | 2006 | yes | yes | yes |  | yes | yes | yes |
| Niemann | 2008 | yes | yes |  |  |  |  |  |
| Noguchi | 2005 | yes |  |  | yes |  |  |  |
| Numans | 2004 | yes |  |  |  |  |  | yes |
| Ogilvie | 2005 | yes |  | yes |  |  |  | yes |
| Ola | 2003 | yes |  | yes |  |  |  | yes |
| Owens | 1996 | yes | yes | yes | yes | yes |  | yes |
| Pai | 2007 | yes |  | yes |  | yes |  | yes |
| Pai | 2005 | yes |  | yes |  | yes |  | yes |
| Pai | 2004 | yes |  | yes | yes | yes |  | yes |
| Pai | 2003 | yes |  | yes |  |  |  |  |
| Pakos | 2007^a^ | yes | yes |  |  | yes |  | yes |
| Pakos | 2007^b^ | yes | yes |  |  | yes |  | yes |
| Pakos | 2005 | yes |  |  |  |  |  | yes |
| Patwardhan | 2004 | yes |  | yes | yes | yes |  | yes |
| Peters | 2008 | yes |  | yes | yes |  | yes | yes |
| Pfeiffer | 2006 | yes |  | yes |  |  | yes | yes |
| Pirozzo | 2003 | yes | yes | yes |  |  |  | yes |
| Price | 2005 | yes |  |  | yes |  | yes |  |
| Puli | 2008^a^ | yes |  | yes |  | yes |  |  |
| Puli | 2008^b^ | yes |  | yes |  | yes |  |  |
| Puli | 2007 | yes |  | yes |  | yes |  | yes |
| Puli | 2008^d^ | yes |  | yes |  | yes |  | yes |
| Puli | 2008^c^ | yes |  | yes |  | yes |  |  |
| Purkayesastha | 2007^b^ | yes |  | yes |  |  |  | yes |
| Purkayesastha | 2007^a^ | yes |  | yes |  |  |  | yes |
| Purkayesastha | 2006 | yes |  | yes |  |  |  | yes |
| Purkayesastha | 2005 | yes |  | yes |  |  |  | yes |
| Reese | 2006 | yes |  | yes |  | yes |  | yes |
| Roddam | 2005 | yes |  | yes |  |  |  | yes |
| Rodgers | 2006 | yes | yes | yes |  | yes |  | yes |
| Ross | 2000 | yes |  |  |  |  |  | yes |
| Royes | 2005 | yes |  | yes |  |  | yes | yes |
| Safdar | 2005 | yes |  | yes |  |  | yes |  |
| Samson | 2002 | yes |  | yes | yes |  |  |  |
| Sarmiento | 2003 | yes |  |  |  |  |  | yes |
| Sauerland | 2004 | yes |  | yes |  |  |  |  |
| Scholten | 2003 | yes |  |  |  |  |  |  |
| Scholten | 2001 | yes |  |  |  | yes |  |  |
| Schreiber | 2003 | yes |  | yes |  |  |  |  |
| Selman | 2005 | yes |  | yes |  | yes | yes | yes |
| **STUDY** |  | **A** | **B** | **C** | **D** | **E** | **F** | **G** |
| Selman | 2008^b^ | yes | yes | yes |  | yes | yes | yes |
| Selman | 2008^a^ | yes | yes | yes |  |  | yes |  |
| Shafiq | 2005 | yes |  |  |  | yes |  |  |
| Shaheen | 2007^b^ | yes |  | yes | yes | yes |  | yes |
| Shaheen | 2007^a^ | yes |  | yes | yes |  |  | yes |
| Shaheen | 2008 | yes |  | yes |  | yes |  |  |
| Shi | 2008 | yes |  | yes |  | yes |  | yes |
| Shie | 2008 | yes |  | yes | yes |  |  | yes |
| Shiga | 2006 | yes |  |  | yes |  | yes | yes |
| Song | 2005 | yes |  |  | yes |  |  |  |
| Sosna | 2008 | yes |  | yes |  |  |  | yes |
| Sotiriadis | 2003 | yes |  |  | yes |  |  |  |
| Speight | 2006 | yes | yes | yes | yes |  |  | yes |
| St John | 2006 |  |  |  |  | yes |  | yes |
| Stein | 2004 | yes |  |  | yes | yes | yes | yes |
| Stein | 2006 | yes |  |  |  |  |  |  |
| Steingart | 2006 | yes |  | yes |  |  |  | yes |
| Steingart | 2007^a^ | yes |  | yes | yes | yes |  | yes |
| Steingart | 2007^b^ | yes |  | yes |  | yes | yes | yes |
| Stengel | 2005 | yes | yes | yes |  | yes | yes | yes |
| Stengel | 2001 | yes |  | yes |  |  |  |  |
| Takata | 2003 | yes |  | yes |  | yes | yes |  |
| Tang | 2007 | yes |  | yes |  |  | yes | yes |
| Terasawa | 2004 | yes |  |  |  |  | yes |  |
| Termaat | 2005 | yes | yes | yes | yes |  |  | yes |
| Tew | 2005 | yes | yes |  |  |  |  |  |
| Trochez-Martinez | 2007 | yes |  | yes | yes |  |  |  |
| Trowbridge | 2003 | yes |  | yes |  |  |  | yes |
| Tse | 2008 | yes |  | yes |  | yes |  | yes |
| Tuon | 2007 | yes |  |  | yes |  |  | yes |
| Tuon | 2006 | yes |  | yes |  | yes | yes | yes |
| Vakil | 2006 | yes |  | yes |  | yes |  | yes |
| van Dongen | 2007 | yes | yes | yes |  | yes | yes | yes |
| van Randen | 2008 | yes | yes | yes | yes | yes |  | yes |
| van Westreenen | 2004 | yes |  | yes |  | yes |  |  |
| van Zaane | 2008 |  | yes | yes |  |  |  | yes |
| Vanezis | 2008 | yes |  | yes |  |  |  |  |
| Vanhoenacker | 2007 | yes | yes | yes |  |  | yes |  |
| Vasbinder | 2001 | yes |  | yes | yes |  |  |  |
| Vestergaard | 2008 | yes |  | yes | yes |  |  |  |
| Virgili | 2007 | yes | yes |  |  | yes |  |  |
| Vlaar | 2007 | yes |  | yes |  |  | yes |  |
| von Roon | 2007 | yes |  | yes |  |  |  | yes |
| Vroomen | 1999 | yes |  | yes |  |  |  |  |
| Wang | 2006 | yes |  | yes | yes | yes | yes | yes |
| **STUDY** |  | **A** | **B** | **C** | **D** | **E** | **F** | **G** |
| Wang | 2005 | yes |  | yes | yes | yes |  |  |
| Wang | 2008 | yes |  | yes |  | yes |  | yes |
| Wardlaw | 2006 | yes | yes |  |  | yes |  | yes |
| White | 2000 | yes |  |  | yes |  |  | yes |
| Whiting | 2006^b^ | yes | yes | yes |  | yes |  | yes |
| Whiting | 2006^a^ | yes |  | yes |  | yes |  |  |
| Whitsel | 2000 | yes |  |  | yes |  |  |  |
| Will | 2006 | yes |  | yes |  |  |  | yes |
| Williams | 2007 | yes |  |  |  | yes | yes | yes |
| Wittkampf | 2007 | yes |  | yes | yes | yes | yes | yes |
| Worster | 2002 | yes |  |  | yes | yes |  |  |
| Worster | 2008 | yes |  | yes |  | yes | yes |  |
| Wyeskes | 2004 | yes |  |  |  | yes | yes |  |

Notes.

In addition to PRISMA, data was also extracted under the following column headings

A Study objectives were clearly stated

B Study reported a search algorithm

C Study reported searching more than 2 electronic databases

D Study reported the inclusion criteria as an algorithm

E Study reported that 2 investigators independently screened, selected, abstracted data from studies

F The Inclusion/exclusion decisions made explicit as a flowchart

G Data abstraction: studies clearly reported the method and data items collected
